# Consequences and opportunities arising due to sparser single-cell RNA-seq datasets

**DOI:** 10.1186/s13059-023-02933-w

**Published:** 2023-04-21

**Authors:** Gerard A. Bouland, Ahmed Mahfouz, Marcel J. T. Reinders

**Affiliations:** 1 Delft Bioinformatics Lab, Delft University of Technology, Delft, The Netherlands; 2 Department of Human Genetics, Leiden University Medical Center, Leiden, 2333ZC The Netherlands; 3 Leiden Computational Biology Center, Leiden University Medical Center, Leiden, 2333ZC The Netherlands

## Abstract

With the number of cells measured in single-cell RNA sequencing (scRNA-seq) datasets increasing exponentially and concurrent increased sparsity due to more zero counts being measured for many genes, we demonstrate here that downstream analyses on binary-based gene expression give similar results as count-based analyses. Moreover, a binary representation scales up to ~ 50-fold more cells that can be analyzed using the same computational resources. We also highlight the possibilities provided by binarized scRNA-seq data. Development of specialized tools for bit-aware implementations of downstream analytical tasks will enable a more fine-grained resolution of biological heterogeneity.

**Supplementary Information:**

The online version contains supplementary material available at 10.1186/s13059-023-02933-w.

## Background

Since its introduction, single-cell RNA sequencing (scRNA-seq) has been vital in investigating biological questions that were previously impossible to answer [1–4]. Continuous technological innovations are resulting in a consistent increase in the number of cells and molecules being measured in a single experiment. However, at the same time, datasets appear to become sparser, i.e., more zero measurements across the whole dataset. The sparsity has generally been seen as a problem, especially since standard count distribution models (e.g., Poisson) do not account for the excess of zeros [5–8]. This sparked discussions about whether the excess of zeros can be explained by mainly technological or biological factors [5, 8–10]. Jiang et al. [8] discuss the “zero-inflation controversy,” in which a distinction is made between a biological zero, indicating the true absence of a transcript, and a non-biological zero, indicating failure of measuring a transcript that was present in the cell. Similarly, Sarkar and Stephens [11] make a distinction between measurement and expression. They proposed a model that is a combination of an expression model that encodes the true absence of a transcript, i.e., a (biological) zero, with a measurement model, for which they use a Poisson model (which can result in non-biological zeros due to limited sequencing depth). Consequently, even non-biological zeros encode useful biological information as then the gene is unlikely to be highly expressed. Or, in other words: all zeros in scRNA-seq datasets have biological significance. Aligned with this, Qui et al. [12] proposed to “embrace” all zeros as useful signal and developed a clustering algorithm requiring only binarized scRNA-seq data (a zero representing a zero count and a one for non-zero counts). Using binarized scRNA-seq data, Qui et al. identified clusters similar to clusters identified using a count-based approach. Although this was the first paper explicitly embracing zeros as useful signal, binarization of scRNA-seq was already used to infer gene regulatory networks [13]. Since then, several methods have employed binarized scRNA-seq data. For instance, scBFA [14], a dimensionality reduction method for binarized scRNA-seq data, showed improved visualization and classification of cell identity and trajectory inference when compared to methods that use count data. Likewise, we introduced binary differential analysis (BDA) [15], a differential expression analysis method relying on binarized scRNA-seq data. We showed that differential expression analysis on binary representations of scRNA-seq data faithfully captures biological variation across cell types and conditions.

Provided that a binarized data representation has the potential to reduce required computational resources considerably, and as scRNA-seq datasets are becoming increasingly bigger and sparser, we wondered if binary should be the preferred data representation for other tasks. In this work, we explore the consequences of sparser datasets and the applicability of binarized scRNA-seq data for various single-cell analysis tasks.

## Results and discussion

**Fig. 1:**
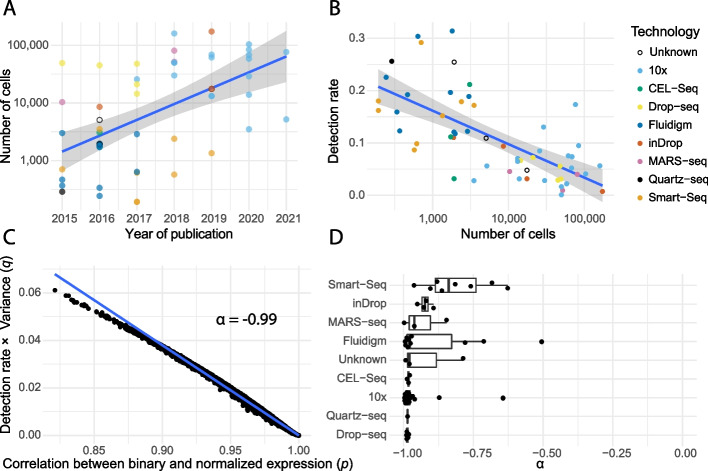
More cells, more zeros. Binarized scRNA-seq datasets were generated by binarizing the raw count matrix, where zero remains zero and every non-zero value is assigned a one. **A** Association between year of publication, total number of cells. Scatterplot of the number of cells (log scale) against the date of publication. **B** Scatterplot of the detection rate (*y*-axis) against the number of cells (log scale, *x*-axis). **C** On the *x*-axis the Pearson’s correlation coefficient (*p*) of every cell from the PaulHSC dataset between the binarized and normalized expressions. On the *y*-axis the product of the detection rate and the variance of the non-zero values (*q*). *α* is the Pearson’s correlation coefficient between these values *p* and *q* across all cells. **D** Boxplots of the *α*-values for all 56 datasets grouped by technology. One dataset (LawlorPancreasData) was excluded as *α*-value (*α* = 0.42) for this dataset was a clear outlier

We downloaded 56 datasets published between 2015 and 2021. Based on these datasets, a clear association between the year of publication and the number of cells can be observed (Pearson’s correlation coefficient of *r* = 0.46, Fig. 1a). For instance, the average dataset in 2015 (*n* = 7) had 704 cells while the average dataset in 2020 (*n* = 7) had 58,654 cells. Another clear trend that can be seen is that an increasing number of cells is highly correlated with decreasing detection rates (fraction of non-zero values) (Pearson’s correlation coefficient of *r* = − 0.47, Fig. 1b). Note that this trend of measuring more cells per dataset outweighs improved chemistry over time and thus still results in sparser datasets. It is likely that this trend will continue over the next years as, for many biological questions, shallow sequencing of many cells is more cost effective than deep sequencing of a few cells [16]. Moreover, by measuring more cells, we can better estimate the probability whether a gene is expressed, and the overall power to detect differentially expressed genes in a given dataset increases [17]. This trend will be amplified, as more population scale and multi-condition scRNA-seq datasets are emerging [17, 18], for which a low coverage sequencing is sufficient to capture cell type specific gene expression (given enough cells are measured per individual and per cell type) [19]. Altogether, these developments will result in sparser scRNA-seq datasets with larger numbers of cells.

As zeros become more abundant, a binarized expression might be as informative as counts. Using ~ 1.5 million cells from 56 datasets, we observed on average a strong point-biserial correlation (Pearson correlation coefficient *p* = 0.93) between the normalized expression counts of a cell and its respective binarized variant, although differences between datasets exist (Additional file 1: Fig. S1). This strong correlation implies that the binarized signal already captures most of the signal present in the normalized count data. This strong correlation is primarily explained by the detection rate (Additional file 1: Fig. S2a) and the variance of the non-zero counts of a cell (Additional file 1: Fig. S2b). In cells where the detection rate is low (many zeros) and the variance of the non-zero counts is small, the correlation between the normalized expression values and their binary representation is high (Fig. 1c). Across all datasets, the detection rate and variance of measured expressions were good predictors for the correlation between the binary representation and the normalized representation, although differences between technologies exist (Fig. 1d). This indicates that as datasets become sparser, counts become less informative with respect to binarized expression.

**Fig. 2:**
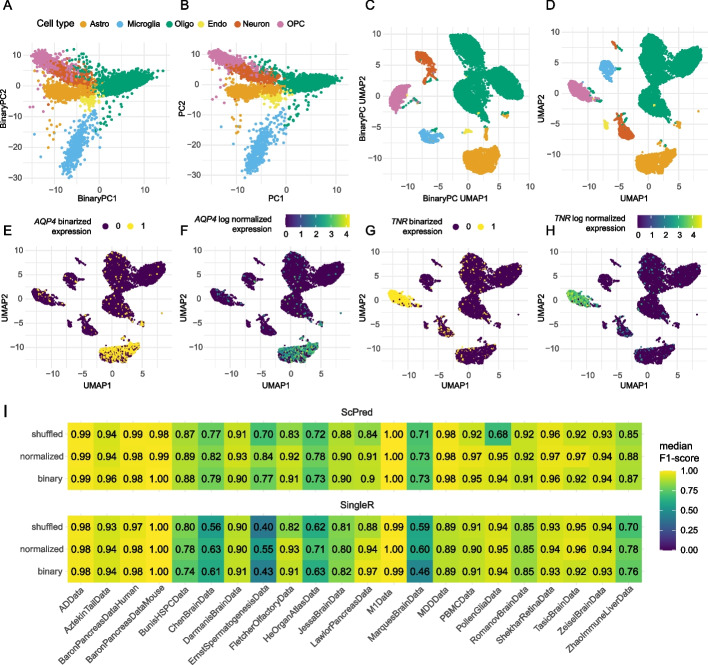
**A**, **B** Cells plotted against the first two principle components of the AD dataset [20]. **A** PCA based on binary representation, and **B** PCA based on count representation. UMAP generated from data presented with **C** the binary-based PCs and **D** the count-based PCs. Colors indicate annotated cell type. **E**, **H** UMAP based on the count based PCs, in which cells are colored according to the binary representation of the marker genes *AQP4* (**E**) and *TNR* (**H**) which are known markers for astrocytes and OPCs respectively [21]. **F**, **G** Similar as **E** and **H** but showing the normalized expression of the marker gene. **I** The performance (median F1-score) of cell type identification by SingleR [22] and scPred [23] when applied to binary (binarized data), normalized (normalized expression), and shuffled (shuffled normalized expression) for 22 datasets

To assess whether counts can actually be discarded in practice, we assessed whether binarized data can give comparable results to counts in four common single-cell analysis tasks: (1) dimensionality reduction for visualization, (2) data integration, (3) cell type identification, and (4) differential expression analysis using pseudobulk. First, for dimensionality reduction, we used three different dimensionality reduction approaches on binarized scRNA-seq data; (i) scBFA [14], (ii) PCA (Fig. 2a), and (iii) eigenvectors of the Jaccard cell–cell similarity matrix (see Additional file 2). All three approaches were compared to the standard approach of applying PCA to the normalized counts (Fig. 2b, Additional file 1: Fig. S3). Further, for all four methods, the first ten components were used to generate a non-linear embedding using UMAP (Additional file 1: Fig. S4). Qualitatively, we observed that the results of binary-based dimensionality reduction are comparable to standard count-based methods. This was confirmed quantitatively, as the pairwise distances between cells based on the binary-based UMAPs were highly correlated with the pairwise distances from the count-based UMAP (*r* ≥ 0.73, Additional file 1: Fig. S5). Especially the UMAP generated with the binary-based PCs was visually very similar to the UMAP generated with the count-based PCs (Fig. 2c, d). Calculating the silhouette score (SS) for each cell type with the reduced dimensions (*n* = 10) resulted in slightly lower scores for scBFA (SS = 0.32) and binary-based PCA (SS = 0.39) compared to the count-based PCA (SS = 0.44) (Additional file 1: Fig. S6). However, in the UMAP space (2-dimensional), silhouette scores for scBFA (SS = 0.43) and binary-based PCA (SS = 0.42) were higher than count-based PCA (SS = 0.35).

Second, we integrated three scRNA-seq datasets [20, 24, 25] with Harmony [26], using count- and binary-based PCA. Both, visually and quantitatively, we observed an improved mixing of cells for the binary representation (LISI = 1.18) as compared to counts (LISI = 1.12) (Additional file 1: Figs. S7 and S8). Third, we evaluated the effect of binarization on cell annotation using (i) marker genes and (ii) classification methods. Using a set of known brain cell type markers [21], we annotated the binarized AD dataset [20] based on solely the detection of respective cell type markers (see Additional file 2). The annotations were compared to cell type labels that were originally assigned based on the markers’ expression level (i.e., counts). We observed a high level of concordance between annotations as quantified by a median F1-score of 0.93. (Additional file 1: Fig. S9). Additionally, we found that the visualization of the binarized expression of cell type markers to be highly similar to the visualization of their normalized expression in UMAP plots (Fig. 2e–h, Additional file 1: Fig. S10). Next, we compared the performance of automatic cell type identification using scPred and SingleR [22, 23] on 22 datasets for which cell type annotations were available. The median F1-scores were highly similar between cell type identifications based on the binarized and the normalized count data, despite large variation of sparseness between these datasets. This finding implies that counts do not add information for cell type identification. This conclusion was further supported by randomly shuffling the non-zero counts, which resulted in a comparable performance (Fig. 2I, Additional file 1: Fig. S11).

Forth, we evaluated whether counts can also be discarded when pseudobulk data is used for differential expression analysis [18]. In a dataset containing scRNA-seq data of the prefrontal cortex of 34 individuals [27], we generated pseudobulk data by either taking the mean expression of each gene across all cells, or the fraction of non-zero values across all cells (detection rate), per individual. Spearman’s rank correlation between the binarized profile and the mean counts (across all genes) was ≥ 0.99 (Additional file 1: Fig. S12) for every individual, implying that pseudobulk aggregation with binarized expression faithfully represents counts. To quantify this further, we generated 960 datasets using muscat [18] with 96 unique simulation settings (see Additional file 2). In each dataset, pseudobulk data for each individual was generated and we identified differential expressed genes using Limma trend [28] for the mean gene expression and a *t*-test for the detection rate. In general, the F1-scores for the count and binary representations were very similar across the different settings; however, with small sample sizes and fewer cells, analyses based on a count representation performed better, while analyses based on a binarized expression performed better with larger sample sizes and more cells (Additional file 1: Fig. S13). Additionally, count-based analyses resulted in more false positives (Additional file 1: Fig. S14), while binarized-based analyses resulted in more false negatives (Additional file 1: Fig. S15). The false negatives were primarily due to highly expressed genes that show no differences in the detection rate. At larger sample sizes and with more cells, the false negatives diminished (Additional file 1: Fig. S16). All together, these result show that most of the information is indeed captured in the binary representation, only when genes have a high detection rate (> 0.9), or when the number of cells per sample becomes low, then, changes in expression are not reflected in the binary representation and, consequently, information from counts is needed.

Whether zero-inflation associates with technical or biological origins is heavily debated [8]. One compelling reason for this debate is the fact that within a single dataset some genes are zero-inflated, while others are not [5, 8]. We argue that this observation is mostly related to whether a gene is only expressed in a subpopulation of cells (e.g., marker genes) or whether a gene has a stable expression (e.g., housekeeping genes). To substantiate our claim, we used BDA [15] to identify the top 100 most differentially expressed genes between two cell populations and the top 100 most stable expressed genes in a 10X dataset [24] as well as a Smart-Seq dataset [29]. Next, we applied scRATE [5] to identify the best distribution model for the observed expression of the identified genes, being either a Poisson, a negative binomial, or their zero-inflated counterparts. A Fisher exact test showed that a zero-inflated model was enriched in the top 100 differentially expressed genes, and a non-zero inflated model was enriched in the top 100 stable expressed genes (Table 1). Hence, like earlier work [5], we conclude biological heterogeneity to be the main driver of zero-inflation.

**Table 1:** Enrichment of zero-inflated distributions for the top100 differential expressed genes and the enrichment of non-zero inflated distributions for the top100 stable genes

Platform	Top 100	Zero-inflated	Not zero-inflated	logOR	95% CI	*P*-value
10x	Differentially expressed genes	99	1	5.19	3.36, 8.87	3.03 × 10^−25^
	Stable genes	35	65			
Smart-seq	Differentially expressed genes	97	3	3.70	2.50, 5.36	5.46 × 10^−18^
	Stable genes	44	56			

Increasingly larger datasets require increasingly more computational resources. The storage required for all 56 datasets used in this study was 764 gigabytes after normalization using sctransform [30] or 276 gigabytes when log-normalized and stored as sparse matrices. In contrast, binarizing the same datasets and storing them as bits required only 73 gigabytes, which is an ~ 11-fold and ~ fourfold reduction in storage requirements, respectively (Additional file 1: Fig. S17). Yet, there are big differences across datasets. For example, a reduction of ~ 50-fold and ~ 20-fold, respectively, was acquired for the BuettnerESC dataset [31]. The amount of storage that can be saved is highly correlated with the detection rate (Additional file 1: Fig. S18), with the highest gain for datasets with a high detection rate. The considerable storage reduction of the binary representation gives the potential to boost downstream analyses to larger numbers of cells, opening possibilities to get a more fine-grained resolution of biological heterogeneity [32].

We showed that analyses based on a binary representation of scRNA-seq data perform on par with count-based analyses. Working with binarized scRNA-seq data has clear additional advantages. The first is simplicity. For the various tasks that we explored, such as dimensionality reduction, data integration, cell type prediction, differential expression analysis [15], and clustering [12], the binary representations required no normalization. Hence, various subjective choices on the normalization could be avoided, which improves reproducibility of these tasks. However, as sequencing depth has an effect on the detection rate of a cell, it is likely this is not the case for all downstream tasks. Second, binarization reduces the amount of required storage significantly and allows the analysis of significantly larger datasets. For example, binary-based data allow for a bit implementation of clustering as has been done before in the field of molecular dynamics resulting in a significant reduction of run time and peak memory usage compared to existing methods [33]. It has also been suggested that binarization alleviates noise [14] as it is insensitive to count errors. However, binarization remains sensitive to detection errors caused by, e.g., the presence of ambient RNA. Consequently, detection of ambient RNA [34] poses a challenge for binary representations when studying individual cells and thus might require specialized methods to be developed.

At first glance, binarizing scRNA-seq data seems to remove signal. However, genes that are highly expressed across cells will not have a lot of zeros, whereas genes that are lowly expressed across cells will have many. This implies we might be able to infer the relative expression of a gene within an individual cell by exploiting the detection pattern of similar other cells. Using this reasoning, we indeed were able to reconstruct the expression levels of genes from the detection pattern using neighboring cells (Additional file 1: Fig. S19, Additional file 2). Hence, we conclude that the detection rate of a gene in a group of cells, such as a cell type, do faithfully represents the (mean) expression levels of that gene in that group of cells, underpinning why binarization for most of the downstream tasks apparently does not have lost signal.

We have shown that sparsity is inversely correlated with the amount of additional signal that is captured with counts. Consequently, binarization will not be useful for all scRNA-seq datasets. Previous work suggested that when the detection rate is > 90%, visualizations based on the binary representation do not perform on par with count-based representation [14]. With our simulation experiments, we have shown a similar trend when considering the task of detecting differential expressed genes based on pseudobulk values.

## Conclusions

Concluding, our results support existing literature in showing that binarized scRNA-seq data can be used for the following: dimensionality reduction, data integration, visualization, clustering, trajectory inference, batch correction, differential expression analysis, and cell type prediction. We believe scRNA-seq tool developers should be aware of the possibility of using a binary representation of the scRNA-seq data instead of count-based data, as it gives opportunities to develop computational- and time-efficient tools.

## Methods

Detailed methods are available in Additional file 2.

## Supplementary Information

Additional file 1: Fig. S1. The distributions of correlation coefficients between the binarized and count-based expressions of every cell. Fig. S2. Dot plots of association between the correlation coefficient between the binarized and count-based representation and detection rate and variance. Fig. S3. Comparison of binary-based dimensionality reductions. Fig. S4. Comparison of binary-based UMAPs. Fig. S5. Association of pairwise Euclidean distances between cells from count based UMAP and binary based UMAP. Fig. S6. Silhouette scores of count- and binary-based dimensionality reduction. Fig. S7. Count- and binary-based UMAP plots of three brain datasets, not integrated. Fig. S8. Count- and binary-based UMAP plots of three brain datasets, integrated. Fig. S9. Heatmap of concordance between binary-based cell type annotations using markers and counts-based cell type annotations using markers. Fig. S10. UMAP plot with expressions of marker genes, using binarized and normalized representations. Fig. S11. Boxplots of the median F1-score of the automatic cell type prediction with different data representations. Fig. S12. Association of detection rate vs mean expression for all genes of one individual. Fig. S13. F1-score on 960 simulated datasets identifying differentially expressed genes in pseudobulk data with either count data or binarized data. Fig. S14. Number of false positives on 960 simulated datasets identifying differentially expressed genes in pseudobulk data with either count data or binarized data. Fig. S15. Number of false negatives on 960 simulated datasets identifying differentially expressed genes in pseudobulk data with either count data or binarized data. Fig. S16. Number of false negatives binned on the detection rate on 960 simulated datasets identifying differentially expressed genes in pseudobulk data with either count data or binarized data. Fig. S17. Storage requirements for the different data representations. Fig. S18. Association of detection rate with fold reduction. Fig. S19. Association between recovered expression and normalized expression.

Additional file 2. Methods.

Additional file 3. Review history.

## Data Availability

All codes, processed data, and analysis results in this paper are publicly available at GitHub [35] and Zenodo [36]. Code used for the analyses and a vignette describing a complete analysis workflow on binarized scRNA-seq data are available on GitHub. The source code is released under the MIT license. The ADData dataset is available at https://www.ncbi.nlm.nih.gov/geo/query/acc.cgi?acc=GSE138852 [37]. The M1Data dataset is available at https://portal.brain-map.org/atlases-and-data/rnaseq/human-m1-10x. The smart-Seq brain dataset available at https://portal.brain-map.org/atlases-and-data/rnaseq/human-mtg-smart-seq. The MDDData dataset is available at https://www.ncbi.nlm.nih.gov/geo/query/acc.cgi?acc=GSE144136 [38]. The PBMCData is available at https://doi.org/10.5281/zenodo.3357167 [39]. All other datasets used in this study are described in the Additional file 2: Table S1 and are available for download from the R-package scRNAseq (v2.8.0) [40].
