# Supplementary material for: Consequences and opportunities arising due to sparser single-cell RNA-seq datasets
Source: Genome Biol. 2023 Apr 21;24:86. doi: 10.1186/s13059-023-02933-w (PMC10120229; doi:10.1186/s13059-023-02933-w)
Supplement: Supplementary file 1 — Additional file 1: Fig. S1. The distributions of correlation coefficients between the binarized and count-based expressions of every cell. Fig. S2. Dot plots of association between the correlation coefficient between the binarized and count-based representation and detection rate and variance. Fig. S3. Comparison of binary-based dimensionality reductions. Fig. S4. Comparison of binary-based UMAPs. Fig. S5. Association of pairwise Euclidean distances between cells from count based UMAP and binary based UMAP. Fig. S6. Silhouette scores of count- and binary-based dimensionality reduction. Fig. S7. Count- and binary-based UMAP plots of three brain datasets, not integrated. Fig. S8. Count- and binary-based UMAP plots of three brain datasets, integrated. Fig. S9. Heatmap of concordance between binary-based cell type annotations using markers and counts-based cell type annotations using markers. Fig. S10. UMAP plot with expressions of marker genes, using binarized and normalized representations. Fig. S11. Boxplots of the median F1-score of the automatic cell type prediction with different data representations. Fig. S12. Association of detection rate vs mean expression for all genes of one individual. Fig. S13. F1-score on 960 simulated datasets identifying differentially expressed genes in pseudobulk data with either count data or binarized data. Fig. S14. Number of false positives on 960 simulated datasets identifying differentially expressed genes in pseudobulk data with either count data or binarized data. Fig. S15. Number of false negatives on 960 simulated datasets identifying differentially expressed genes in pseudobulk data with either count data or binarized data. Fig. S16. Number of false negatives binned on the detection rate on 960 simulated datasets identifying differentially expressed genes in pseudobulk data with either count data or binarized data. Fig. S17. Storage requirements for the different data representations. Fig. S18. Association of detection rate [file 13059_2023_2933_MOESM1_ESM.docx]

**Consequences and opportunities arising due to sparser single-cell RNA-seq datasets**

Gerard A. Bouland^1,2,^, Ahmed Mahfouz^1,2,3,*^, Marcel J.T. Reinders^1,2,3,*^

^1^ Delft Bioinformatics Lab, Delft University of Technology, Delft, The Netherlands

^2^ Department of Human Genetics, Leiden University Medical Center, Leiden 2333ZC, The Netherlands

^3.^Leiden Computational Biology Center, Leiden University Medical Center, Leiden 2333ZC, The Netherlands

*Corresponding authors: Ahmed Mahfouz (a.mahfouz@lumc.nl) and Marcel J.T.Reinders(m.j.t.reinders@tudelft.nl)


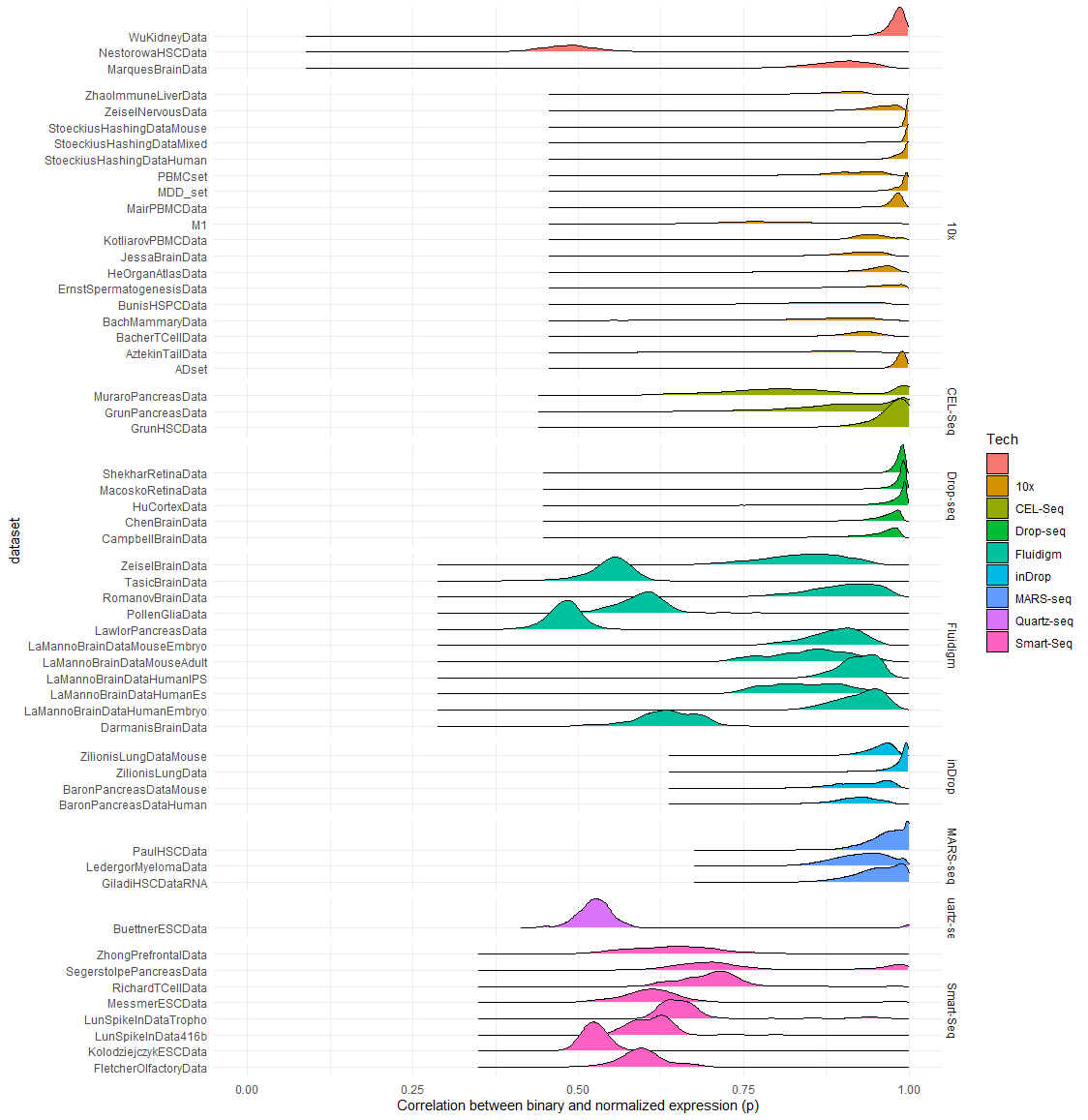


**Fig. S1:** The distributions of correlation coefficients between the binarized and count-based expressions of every cell (p, x-axis) within each dataset (y-axis). The datasets are grouped by technology.


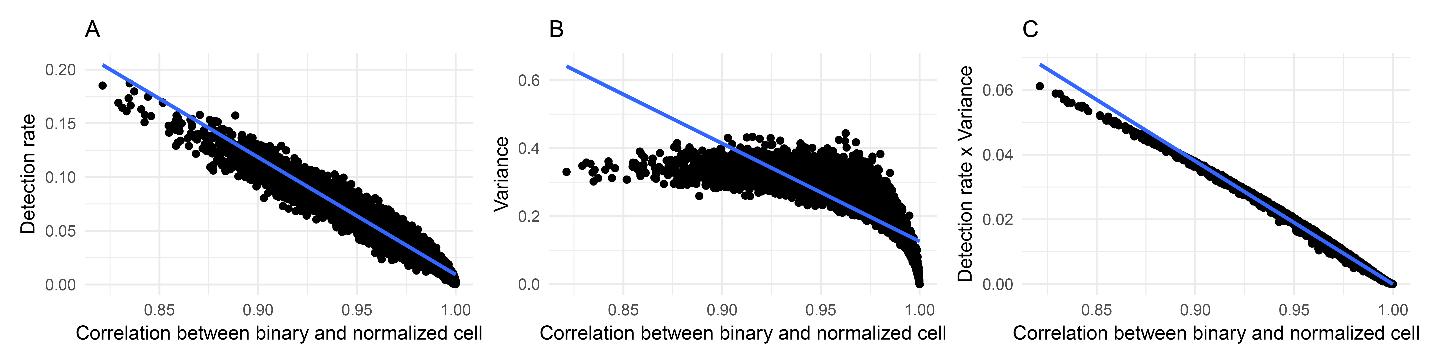
 **Fig. S2: A,B,C)** Every dot is a cell from the PaulHSC dataset. The x-axis represents the correlation coefficient between the binarized and count-based representation. **A)** The y-axis is the detection rate, **B)** the y-axis is the variance of the binarized representation of a gene across all cells, and **C)** the y-axis is the product of detection rate and the variance of non-zero counts.


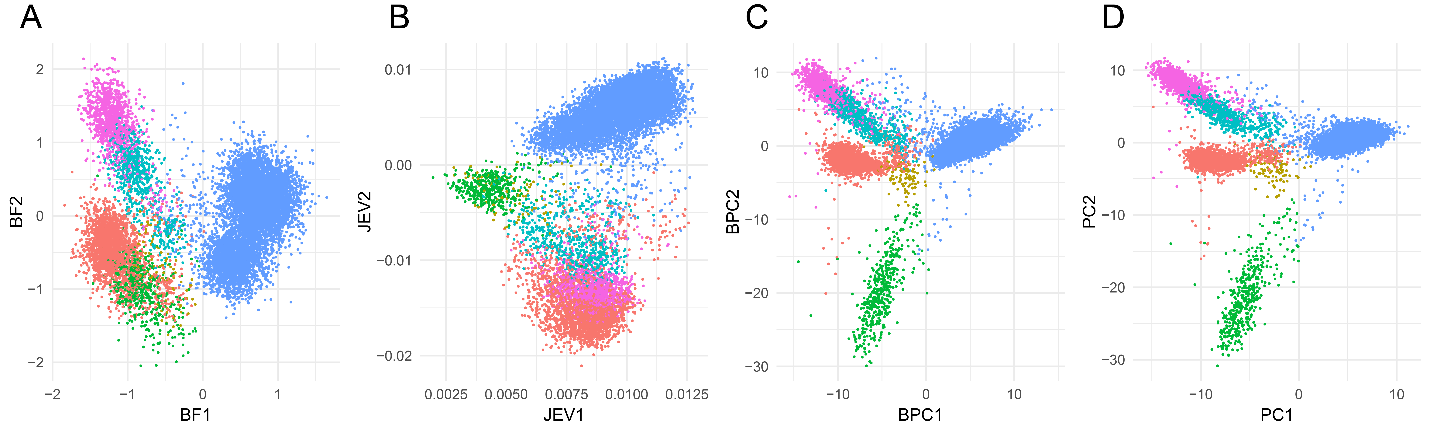


**Fig. S3:** Comparison of binary-based dimensionality reduction on AD Dataset, all points are colored based on pre-annotated cell types. **A)** First two components from scBFA method. **B)** First two components from the Jaccard similarity eigenvectors. **C)** First two components from binary-based PCA. **D)** First two components from count-based PCA.


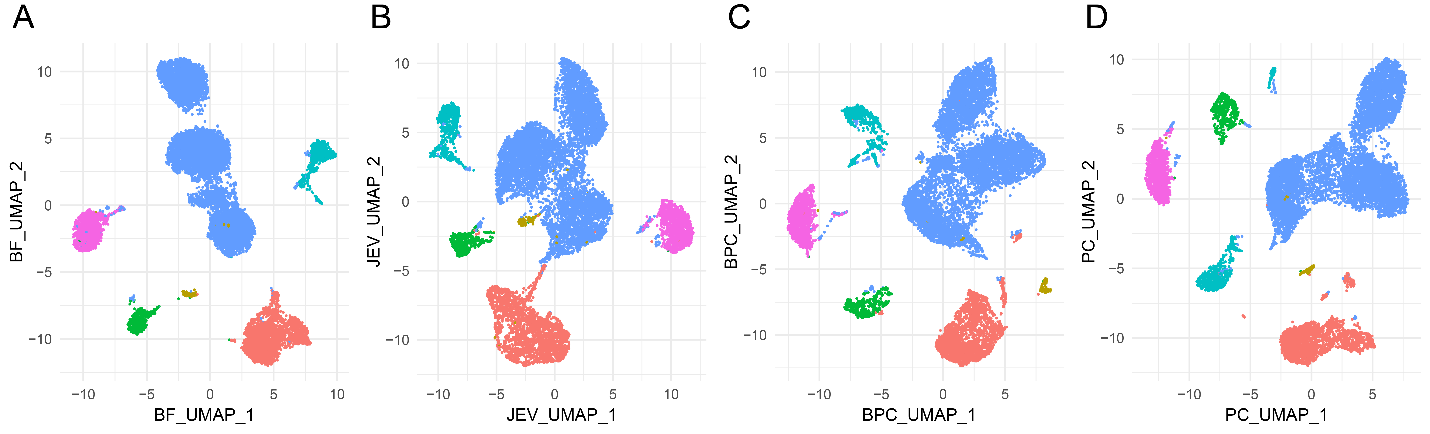


**Fig. S4:** Comparison of binary-based UMAPs on AD Dataset, all points are colored based on pre-annotated cell types. **A)** UMAP plot based on the ten components from scBFA method. **B)** UMAP plot based on the ten components from the Jaccard similarity eigenvectors. **C)** UMAP plot based on the ten components from binary-based PCA. **D)** UMAP plot based on the ten components from count-based PCA.


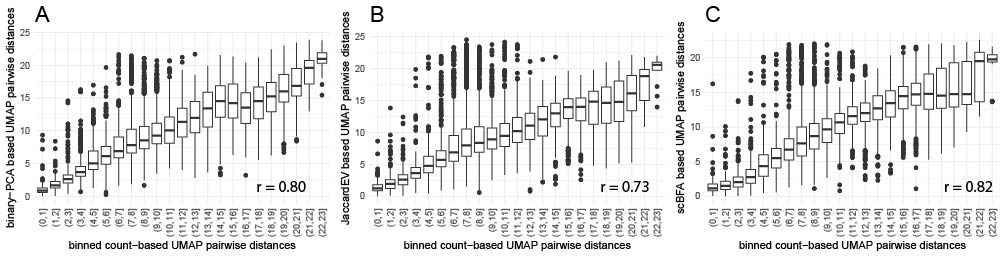


**Fig. S5:** Association of pairwise eucledian distances between cells from count based UMAP with **A)** binary-PCA based UMAP, **B)** JaccardEV based UMAP and **C)** scBFA based UMAP. First, 5,000 cells were randomly sampled, between which the pairwise eucledian distance was calculated based on the different UMAPs. Based on these pair-wise distances (n = 12,497,500) the pearson correlation was calculated. For plotting 10,000 points were randomly sampled from total number of calculated pair-wise distances.


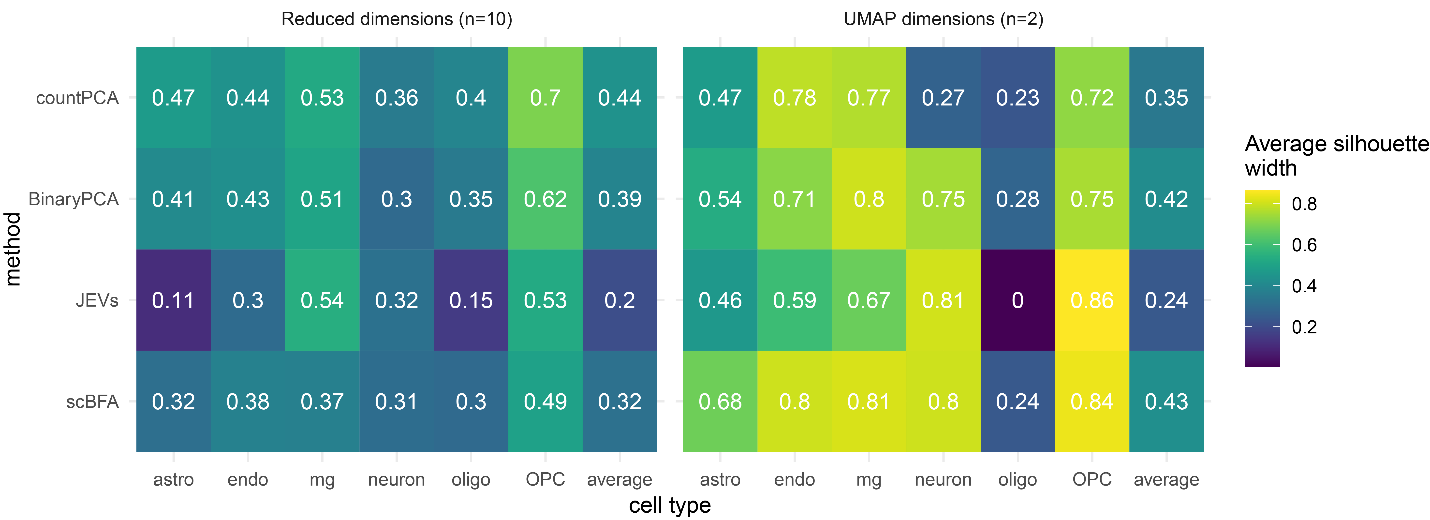


**Fig. S6:** Silhouette scores of count- and binary-based dimensionality reduction. Silhouette scores were calculated with the reduced dimensions and cell types as clusters. CountPCA and BinaryPCA are PCs obtainend with counts and binarized counts repsecitvely. JEVs are Jaccard eigen values and scBFA were components obtained using binary data and scBFA. The last column represents the average of the whole dataset.


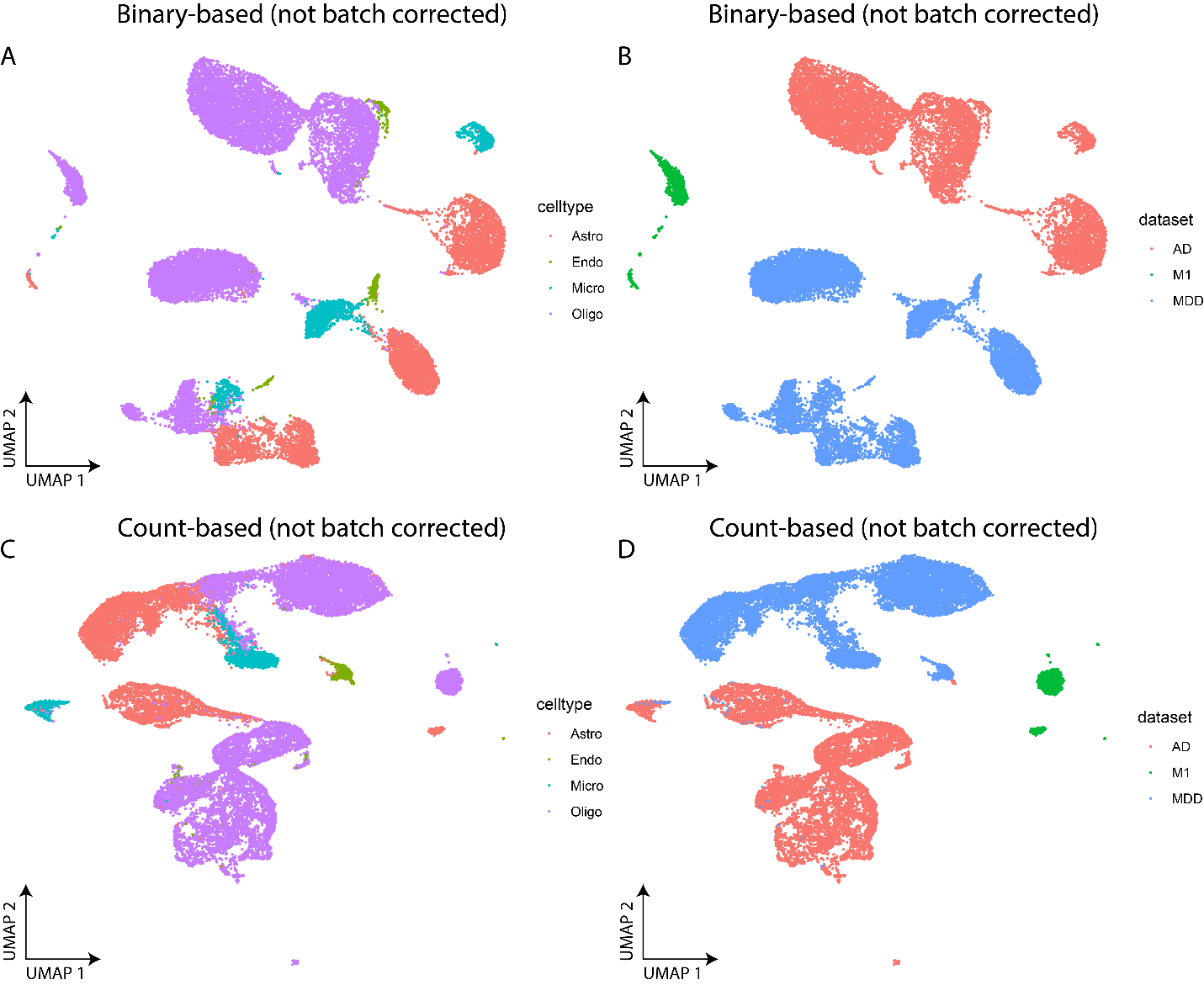


**Fig. S7:** UMAP plots of three brain datasets. **A)** UMAP plot of three brain datasets where the dataset representation was binary, colors indicate cell type. **B)** UMAP plot of three brain datasets where the dataset representation was binary, colors indicate dataset. **C)** UMAP plot of three brain datasets where the dataset representation was log normalized counts, colors indicate cell type. **D)** UMAP plot of three brain datasets where the dataset representation was log normalized counts, colors indicate dataset.


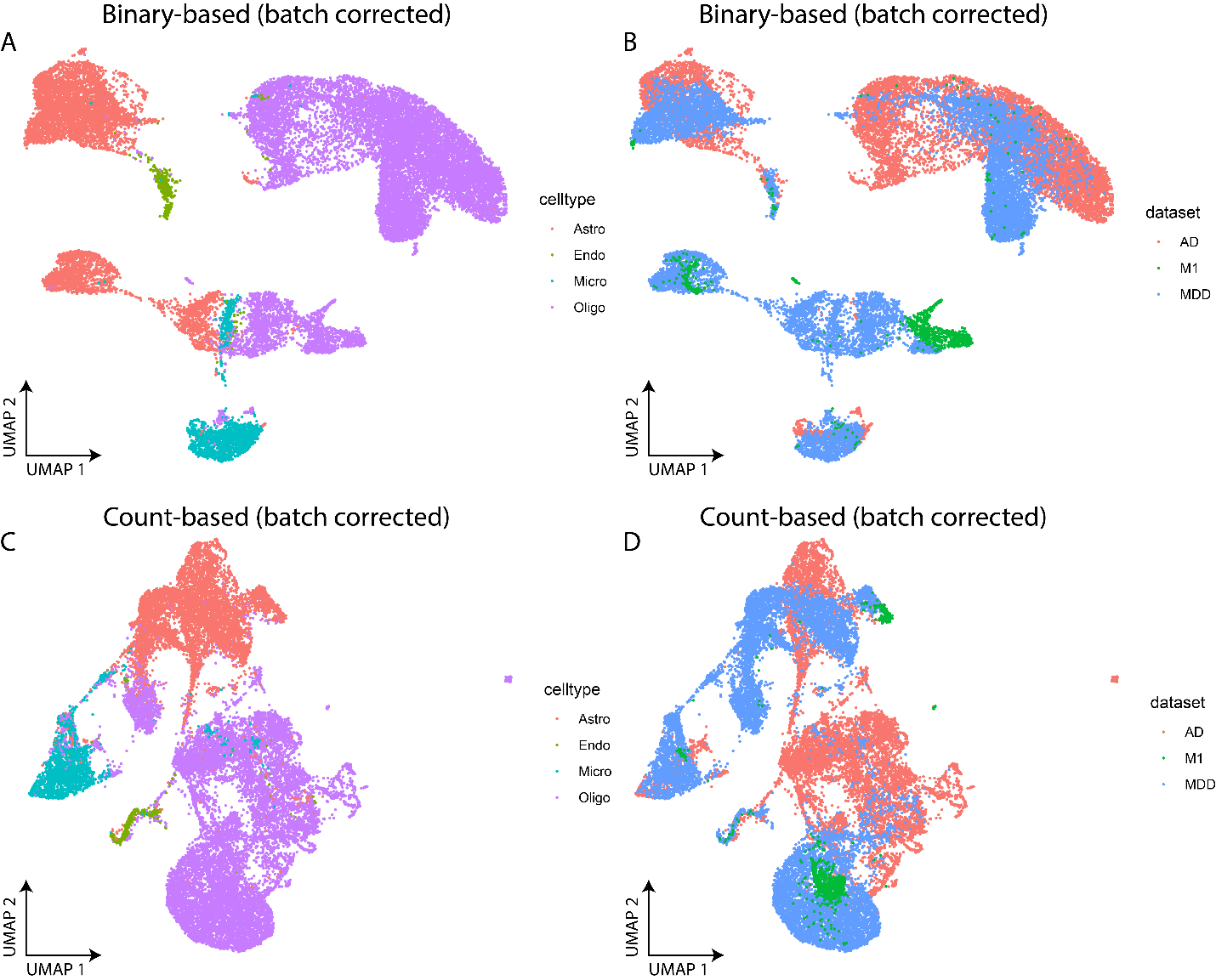


**Fig. S8:** UMAP plots of three brain datasets, batch corrected for datasets using Harmony. **A)** UMAP plot of three brain datasets where the dataset representation was binary, colors indicate cell type. **B)** UMAP plot of three brain datasets where the dataset representation was binary, colors indicate dataset. **C)** UMAP plot of three brain datasets where the dataset representation was log normalized counts, colors indicate cell type. **D)** UMAP plot of three brain datasets where the dataset representation was log normalized counts, colors indicate dataset.

**
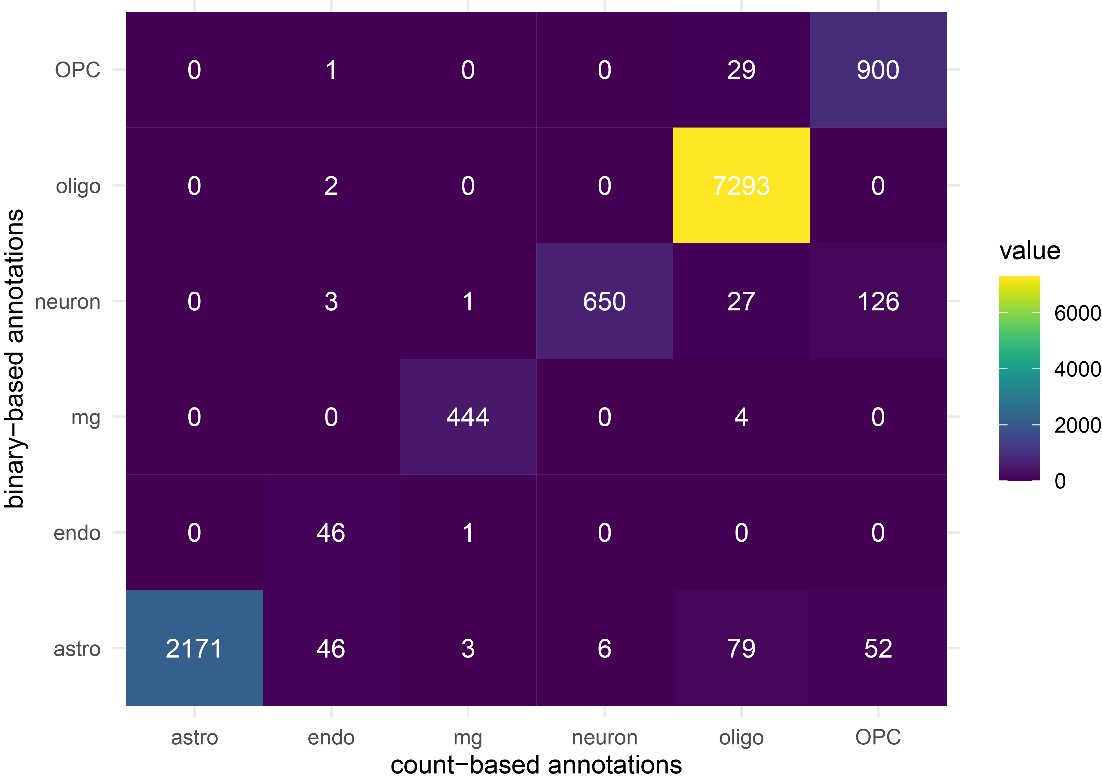
**

**Fig. S9:** Heatmap of concordance between binary-based cell type annotations using markers and counts-based cell type annotations using markers.


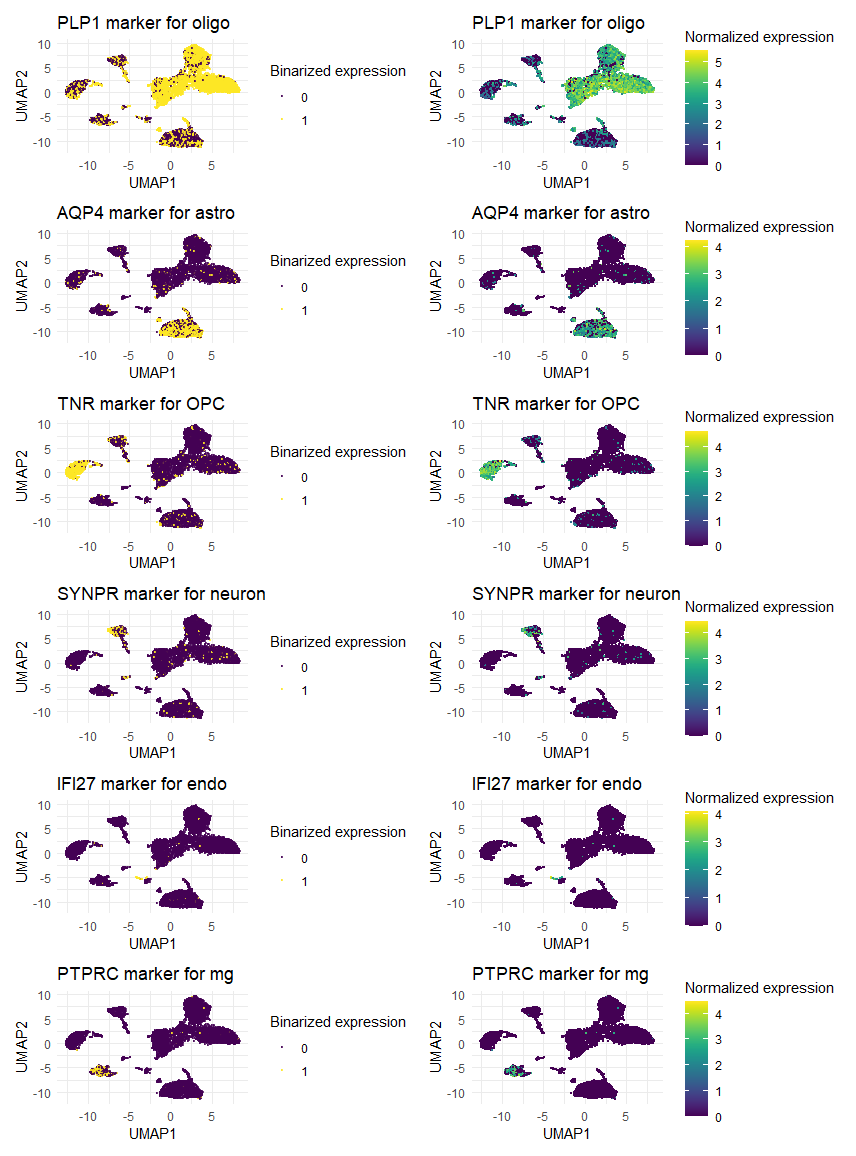


**Fig. S10:** UMAP plot of the AD Dataset with expressions of marker genes, using binarized and normalized representations.

**
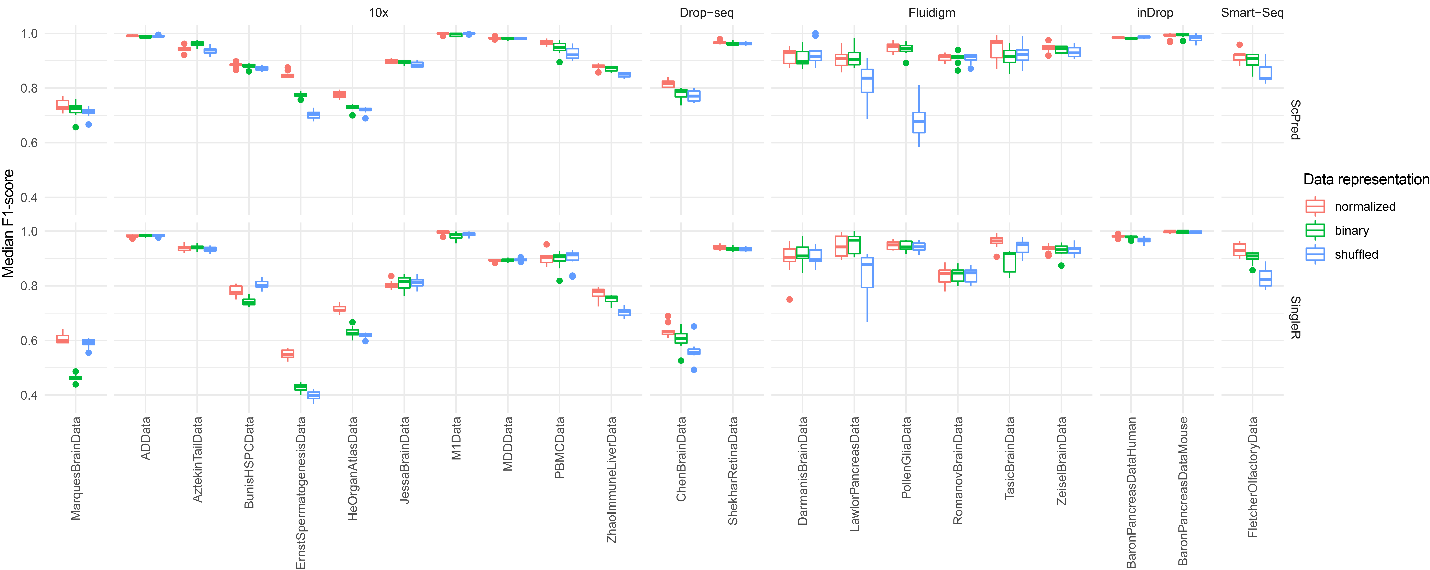
**

**Fig. S11:** Boxplots of the median F1-score of the automatic cell type prediction with different data representations. Both methods (scPred, SingleR) were applied 10 times on each dataset with different reference/target splits. The datasets are represented on the x-axis and the y-axis are median F1-scores.


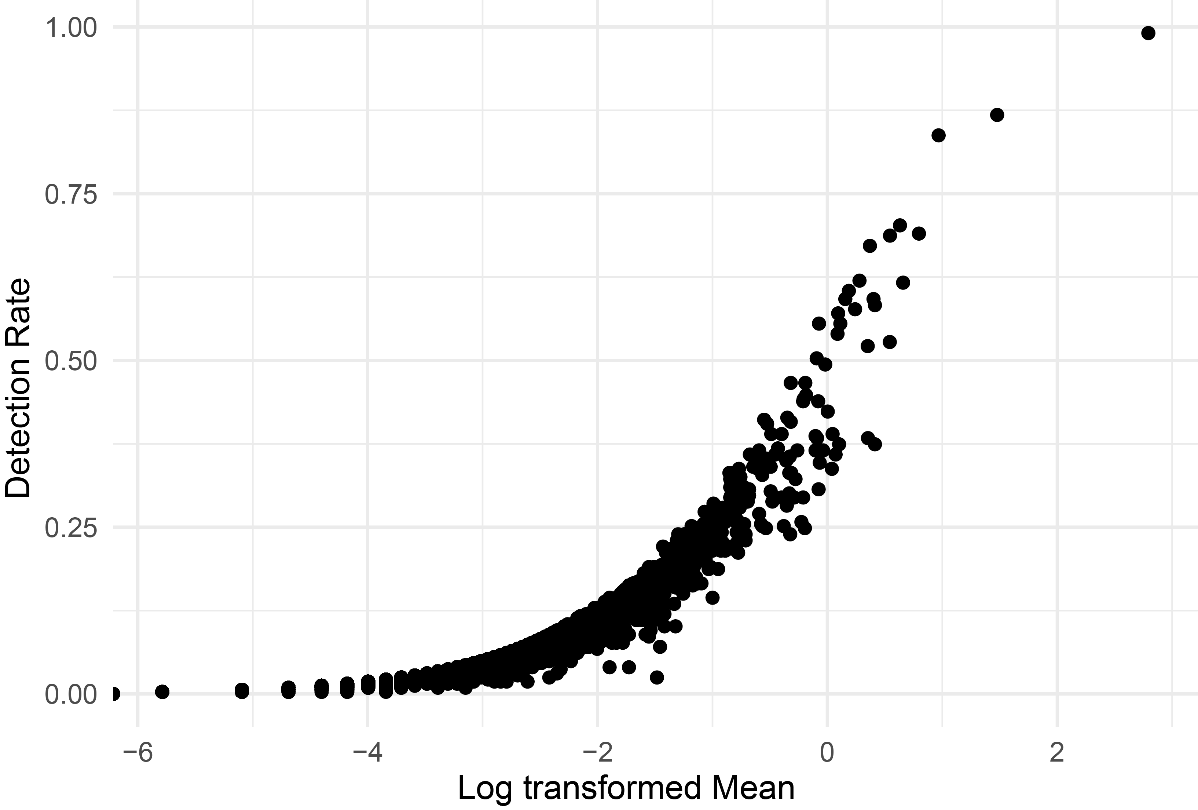


**Fig. S12:** Scatterplot of detection rate (y-axis) vs mean expression(x-axis) for all genes (n = 30.062) of one individual. The Spearman's rank correlation (across all genes) was ≥ 0.99 for all individuals. Note, spearman’s rank correlation was used as this association between detection rate and log transformed mean is known to be non-linear, but their ranks are linearly correlated.


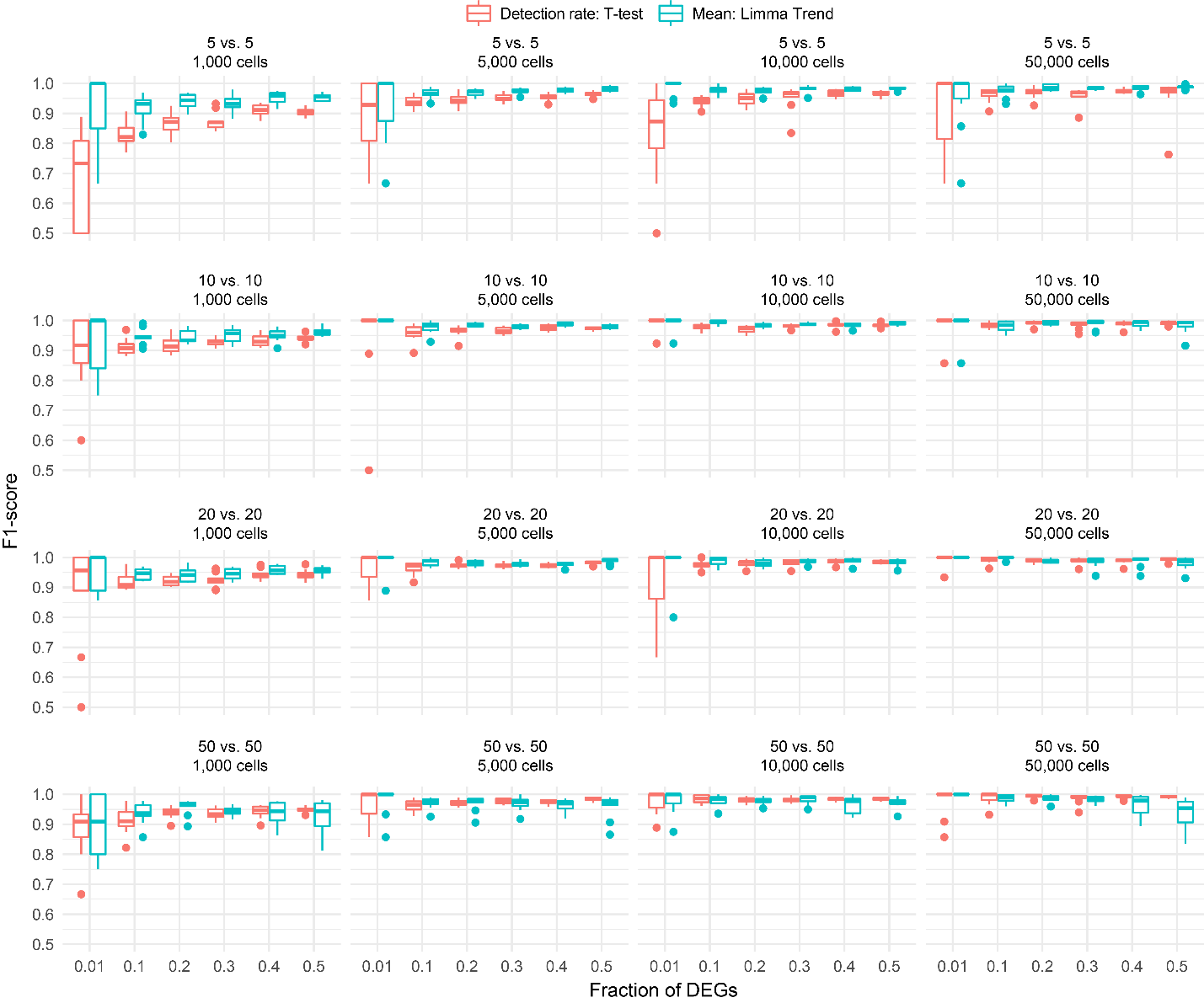


**Fig. S13:** F1-score of 960 simulated datasets indicating the accuracy of detecting differentially expressed genes in simulated pseudobulk data when either count or binarized data are used. The x-axis represents the fraction of simulated differentially expressed genes. The y-axis represents the F1-score. The top-left panel represents a comparison of 5 vs. 5 samples in a simulated dataset of 1.000 cells, meaning that each sample was comprised of 100 cells. E.g in the bottom left panel each sample was comprised of 10 cells.


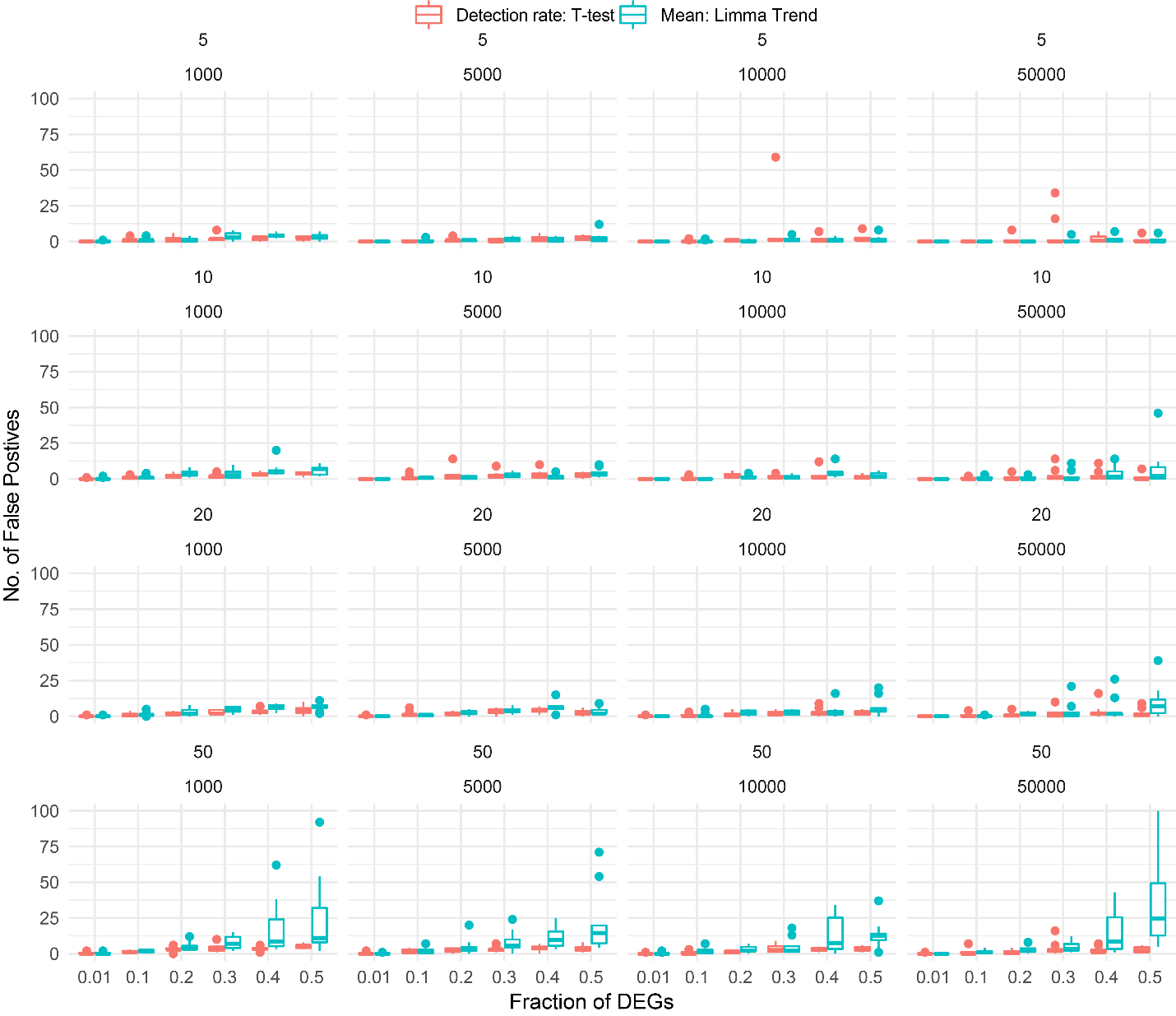


**Fig. S14:** FPR of 960 simulated datasets indicating the accuracy of detecting differentially expressed genes in simulated pseudobulk data when either count or binarized data are used. The x-axis represents the fraction of simulated differentially expressed genes. The y-axis represents the FPR. The top-left panel represents a comparison of 5 vs. 5 samples in a simulated dataset of 1.000 cells, meaning that each sample was comprised of 100 cells. E.g in the bottom left panel each sample was comprised of 10 cells.


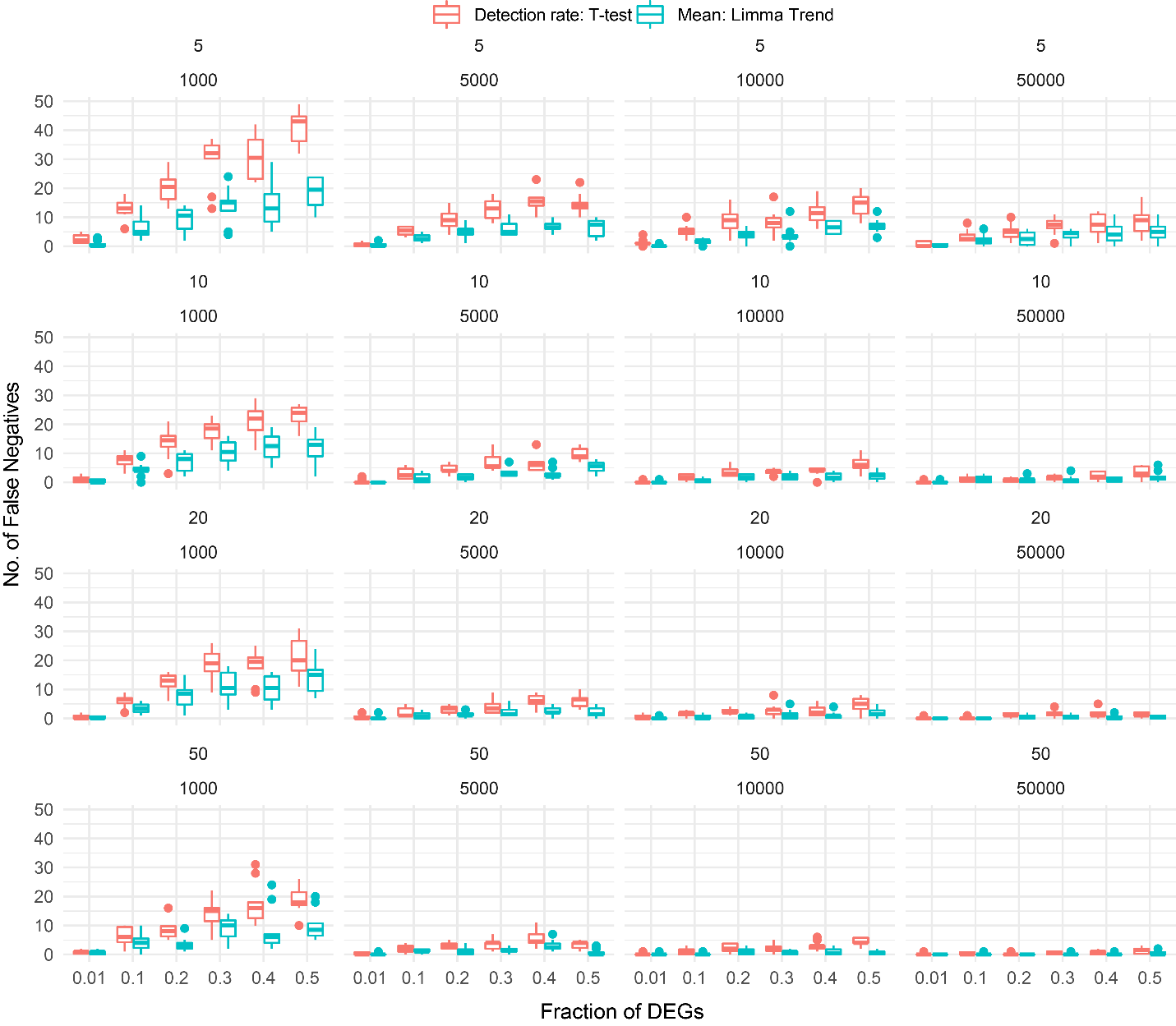


**Fig. S15:** FNR of 960 simulated datasets indicating the accuracy of detecting differentially expressed genes in simulated pseudobulk data when either count or binarized data are used. The x-axis represents the fraction of simulated differentially expressed genes. The y-axis represents the FNR. The top-left panel represents a comparison of 5 vs. 5 samples in a simulated dataset of 1.000 cells, meaning that each sample was comprised of 100 cells. E.g in the bottom left panel each sample was comprised of 10 cells.


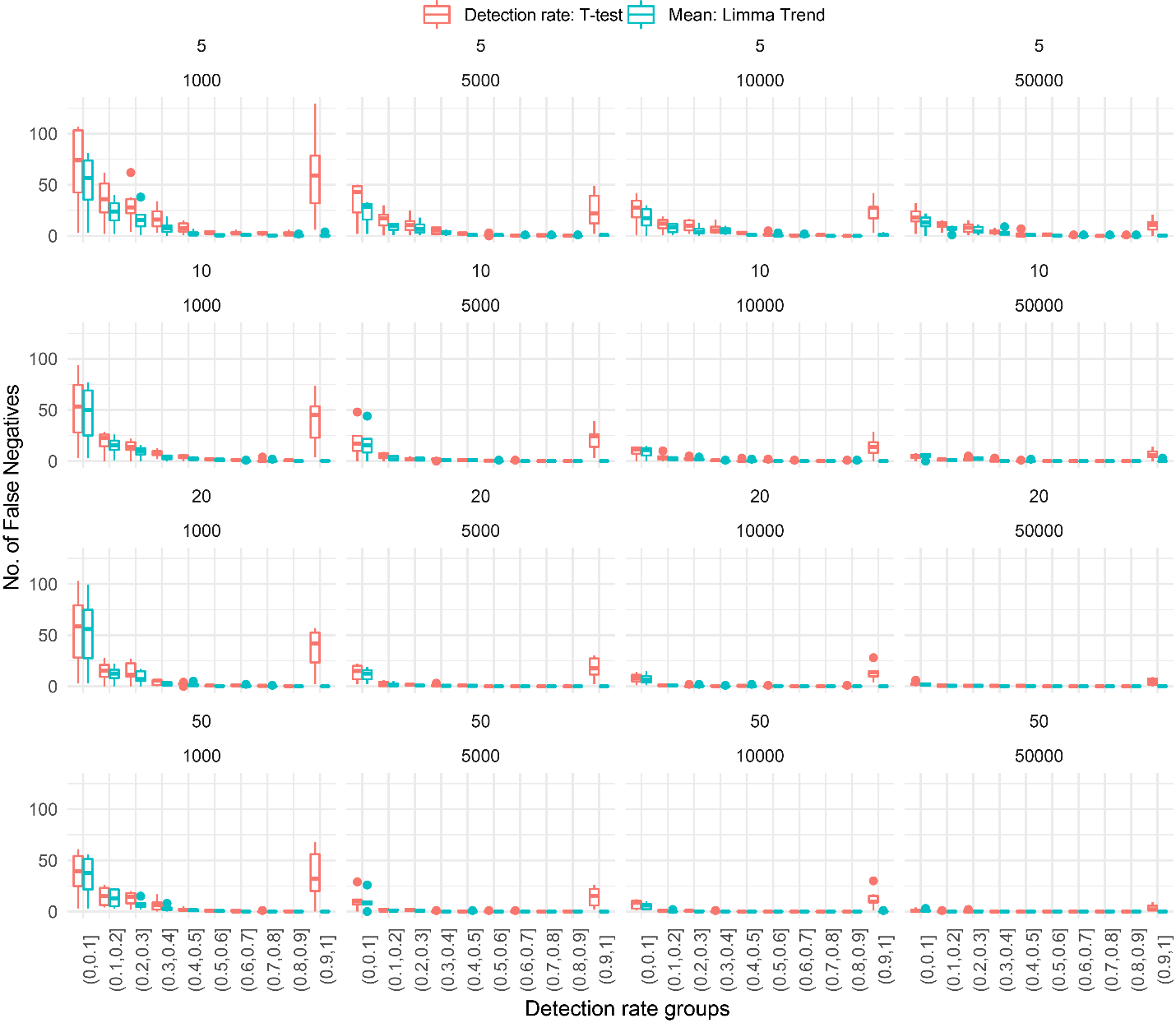


**Fig. S16:** Number of false negatives of 960 simulated datasets indicating how well simulated differentially expressed genes in pseudobulk data can be found back when either count data is used or binarized data. The x-axis represent the detection rate groups. E.g. simulated genes with a detection rate between 0 and 0.1 belong the first group (0,0.1]. The y-axis represent the number of false negatives.


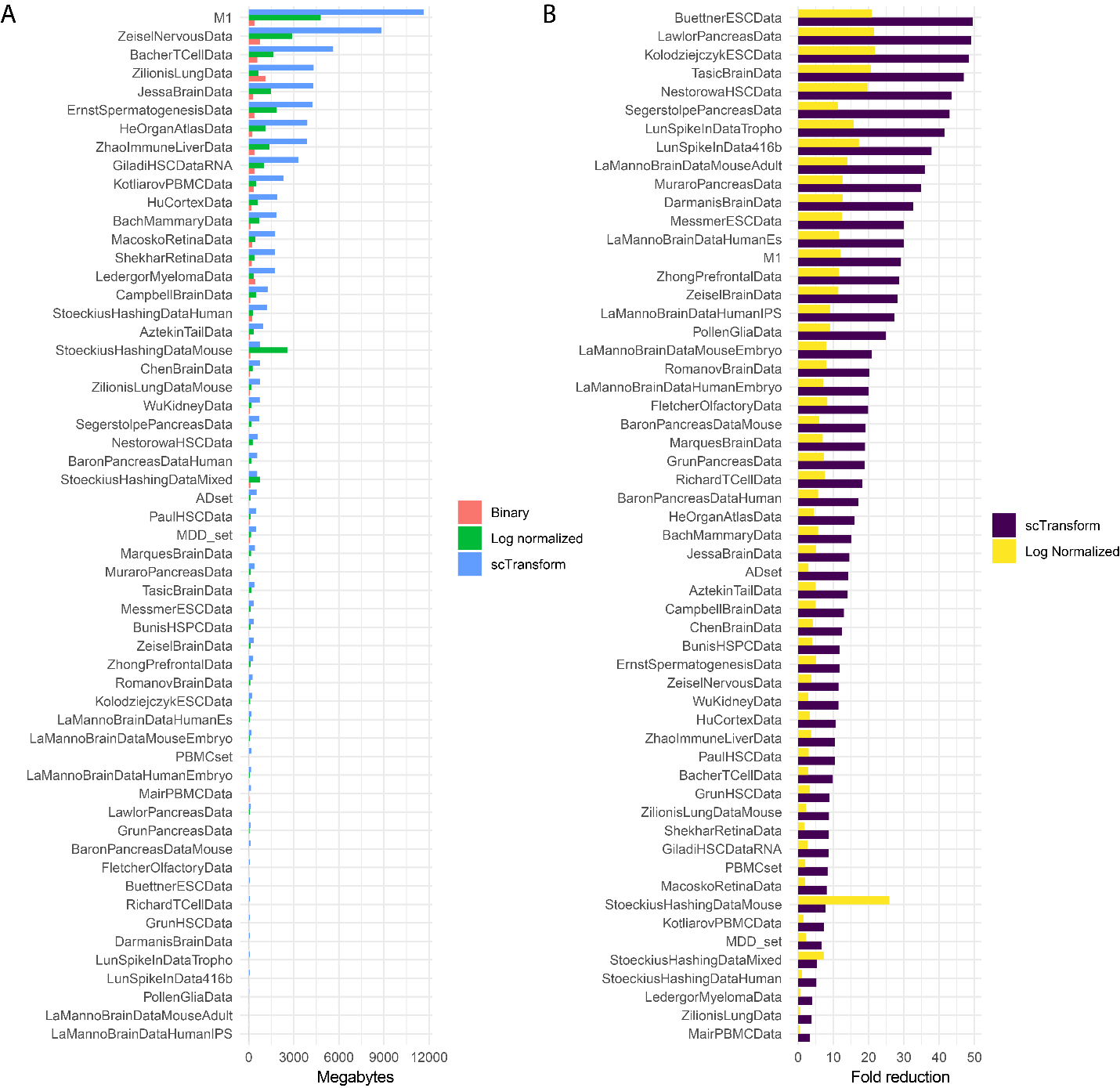


**Fig. S17:** Storage requirements for the different data representations. **A)** For each dataset (y-axis) the required storage required in megabytes (x-axis). **B)** Fold reduction(x-axis) for all datasets(y-axis). Fold reduction of bit-stored relative to scTransform is purple. Fold reduction of bit-stored relative to log normalized is yellow.


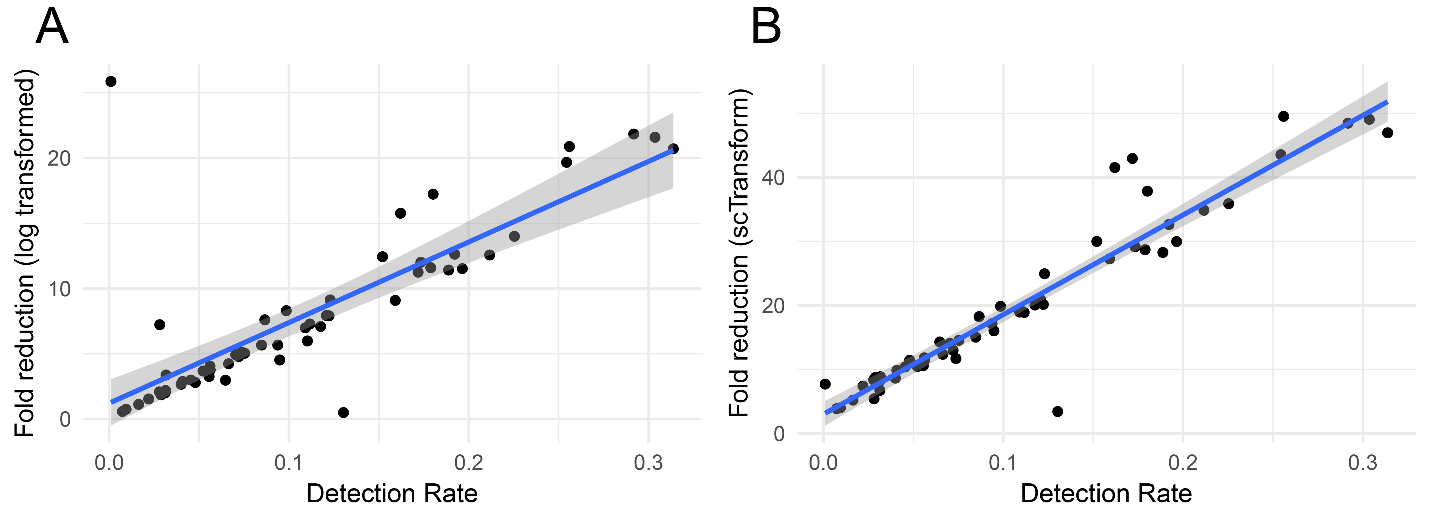


**Fig. S18:** Association of detection rate with fold reduction. Scatter plost where each dot is a dataset, the x-axis represents the detection rate and the y-axis is the fold reduction of bit-stored relative to **A)** log normalized and **B)** scTransform.


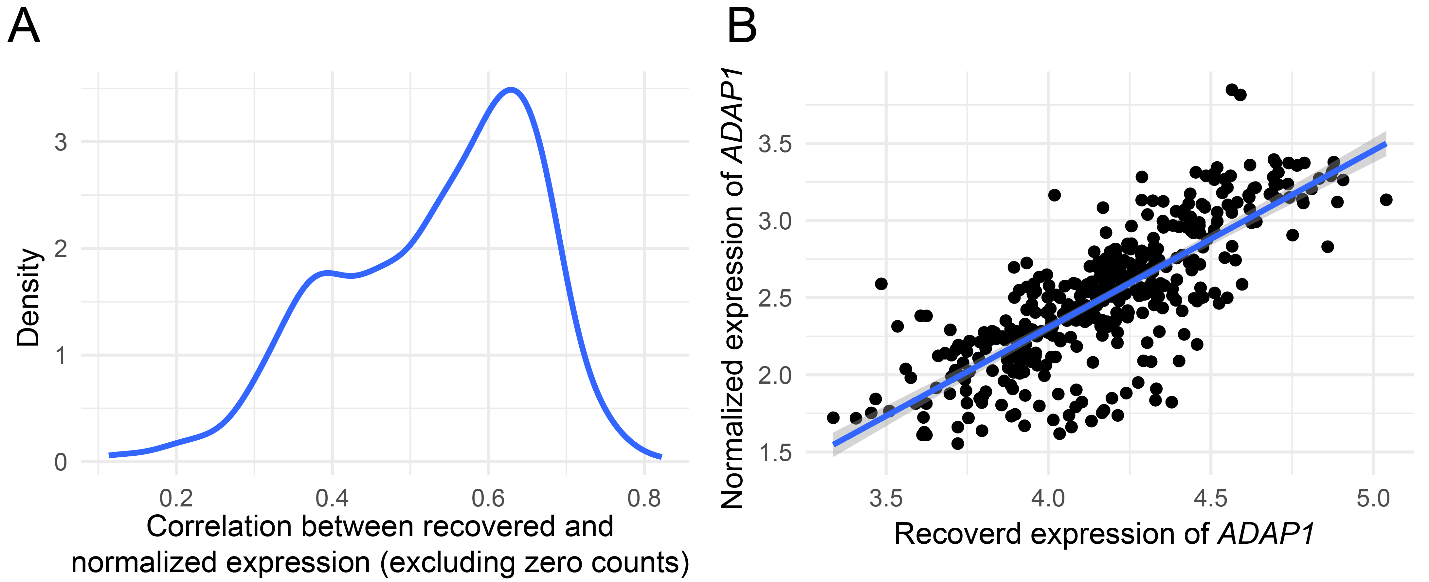


**Fig. S19: A)** Density plot of the correlation coeffiecient between recovered expression values and normalized expression values of the non-zero counts. **B)** Scatter plot showing the recovered expression of *ADAP1* (x-axis), and the normalized expession values of *ADAP1* (y-axis) from the AD dataset. All zero counts are excluded, as these artifically inflate the correlation coefficient.
