# Supplementary material for: Consequences and opportunities arising due to sparser single-cell RNA-seq datasets
Source: Genome Biol. 2023 Apr 21;24:86. doi: 10.1186/s13059-023-02933-w (PMC10120229; doi:10.1186/s13059-023-02933-w)
Supplement: Supplementary file 2 — Additional file 2. Methods. [file 13059_2023_2933_MOESM2_ESM.docx]

**Consequences and opportunities arising due to sparser single-cell RNA-seq datasets**

Gerard A. Bouland^1,2,^, Ahmed Mahfouz^1,2,3,*^, Marcel J.T. Reinders^1,2,3,*^

^1^ Delft Bioinformatics Lab, Delft University of Technology, Delft, The Netherlands

^2^ Department of Human Genetics, Leiden University Medical Center, Leiden 2333ZC, The Netherlands

^3.^Leiden Computational Biology Center, Leiden University Medical Center, Leiden 2333ZC, The Netherlands

*Corresponding authors: Ahmed Mahfouz (a.mahfouz@lumc.nl) and Marcel J.T.Reinders(m.j.t.reinders@tudelft.nl)

# **Supplementary Methods**

**Datasets**

A total of 56 scRNA-seq datasets were used of which 52 datasets were downloaded using the scRNA-seq R-package (v 2.8.0). Four additional datasets were acquired from the corresponding sources (**Table S1**).

**Table S1:** Overview of datasets

| **Name** | **Description** | **Number** | **Tech** | **Reference** |
| --- | --- | --- | --- | --- |
| AztekinTailData | tail | 13199 | 10x | (1) |
| BachMammaryData | mammary gland | 25806 | 10x | (2) |
| BacherTCellData | T cells | 104417 | 10x | (3) |
| BaronPancreasData(HumanMouse) | pancreas | 8569 | inDrop | (4) |
| BaronPancreasData(HumanMouse) | pancreas | 1886 | inDrop | (4) |
| BuettnerESCData | embryonic stem cells | 288 | Quartz-seq | (5) |
| BunisHSPCData | haematopoietic stem and progenitor | 5183 | 10x | (6) |
| CampbellBrainData | brain | 21086 | Drop-seq | (7) |
| ChenBrainData | brain | 14437 | Drop-seq | (8) |
| DarmanisBrainData | brain | 466 | Fluidigm | (9) |
| ErnstSpermatogenesisData | testis | 68937 | 10x | (10) |
| FletcherOlfactoryData | olfactory epithelium | 616 | Smart-Seq | (11) |
| GrunHSCData | haematopoietic stem cells | 1915 | CEL-Seq | (12) |
| GrunPancreasData | pancreas | 1728 | CEL-Seq | (12) |
| GiladiHSCData | haematopoietic stem cells | 81024 | MARS-seq | (13) |
| HeOrganAtlasData | various organs | 84363 | 10x | (14) |
| HuCortexData | cortex | 48000 | Drop-seq | (15) |
| KolodziejczykESCData | embryonic stem cells | 704 | Smart-Seq | (16) |
| JessaBrainData | brain | 61595 | 10x | (17) |
| LaMannoBrainData('human-es') | embryonic stem cells | 1715 | Fluidigm | (18) |
| LaMannoBrainData('human-embryo') | embryonic midbrain | 1977 | Fluidigm | (18) |
| LaMannoBrainData('human-ips') | induced pluripotent stem cells | 337 | Fluidigm | (18) |
| LaMannoBrainData('mouse-adult') | adult dopaminergic neurons | 243 | Fluidigm | (18) |
| LaMannoBrainData('mouse-embryo') | embyronic midbrain | 1907 | Fluidigm | (18) |
| LawlorPancreasData | pancreas | 638 | Fluidigm | (19) |
| LedergorMyelomaData | bone marrow plasma cells | 51840 | MARS-seq | (20) |
| LunSpikeInData('416b') | 416B cells | 192 | Smart-Seq | (21) |
| LunSpikeInData('tropho') | trophoblasts | 192 | Smart-Seq | (21) |
| MacoskoRetinaData | retina | 49300 | Drop-seq | (22) |
| MairPBMCData | peripheral blood mononuclear cells | 29033 | 10x | (23) |
| KotliarovPBMCData | peripheral blood mononuclear cells | 58654 | 10x | (24) |
| MarquesBrainData | brain | 5069 |  | (25) |
| MessmerESCData | embryonic stem cells | 1344 | Smart-Seq | (26) |
| MuraroPancreasData | pancreas | 3072 | CEL-Seq | (27) |
| NestorowaHSCData | haematopoietic stem cells | 1920 |  | (28) |
| PaulHSCData | haematopoietic stem cells | 10368 | MARS-seq | (29) |
| PollenGliaData | outer radial glia | 367 | Fluidigm | (30) |
| RichardTCellData | CD8+ T cells | 572 | Smart-Seq | (31) |
| RomanovBrainData | brain | 2881 | Fluidigm | (32) |
| SegerstolpePancreasData | pancreas | 3514 | Smart-Seq | (33) |
| ShekharRetinaData | retina | 44994 | Drop-seq | (34) |
| StoeckiusHashingData(mode='mouse') | peripheral blood mononuclear cells | 50000 | 10x | (35) |
| StoeckiusHashingData(mode='human') | peripheral blood mononuclear cells | 50000 | 10x | (35) |
| StoeckiusHashingData(type='mixed') | HEK, THP1, K562, KG1 cells | 30000 | 10x | (35) |
| TasicBrainData | brain | 1809 | Fluidigm | (36) |
| WuKidneyData | kidney | 17542 |  | (37) |
| ZeiselBrainData | brain | 3005 | Fluidigm | (38) |
| ZeiselNervousData | nervous system | 160796 | 10x | (39) |
| ZhaoImmuneLiverData | liver immune cells | 68100 | 10x | (40) |
| ZhongPrefrontalData | prefrontal cortex | 2394 | Smart-Seq | (41) |
| ZilionisLungData | lung | 173954 | inDrop | (42) |
| ZilionisLungData('mouse') | lung | 17549 | inDrop | (42) |
| ADData | brain | 13214 | 10x | (43) |
| M1Data | brain | 76533 | 10x | (44) |
| MDDData | brain | 13881 | 10x | (45) |
| PBMCData | peripheral blood mononuclear cells | 3500 | 10x | (46) |

**Binarization and the detection rate**

Binarized scRNA-seq datasets were generated by transforming the raw count matrix such that a zero remains a zero and every non-zero value is assigned a one. The detection rate refers to the fraction of non-zero values. More formally, binarized scRNA-seq data is generated as follows:

$y_{\mathrm{ij}}= \left\{ \begin{matrix} 1 & x_{\mathrm{ij}} \geq1 \\ 0 & \mathrm{otherwise} \end{matrix} , for i\in\left[ 1,g \right], j\in[1,n] \right.$

where $x_{\mathrm{ij}}$ is the expression of gene i in cell j; g is number of genes and n the number of cells in the dataset.

The detection rate $\mathrm{DR}_{j}$ for cell j is then defined as:

$\mathrm{DR}_{j}= \frac{\sum_{i=1}^{g} y_{\mathrm{ij}}}{g}, \forall j\in[1,n]$

Similarly, we define the detection rate for a gene $\mathrm{DR}_{i}$ and the detection rate across the whole dataset $\mathrm{DR}_{d}$:

$\mathrm{DR}_{i}= \frac{\sum_{j=1}^{n} y_{\mathrm{ij}}}{n}, \forall i\in[1,g]$

$\mathrm{DR}_{d}= \frac{\sum_{i=1}^{g} \sum_{j=1}^{n} y_{\mathrm{ij}}}{\mathrm{ng}}$

Note, the detection rate of a gene can be determined either within a specific cell population or within the dataset*.*

**Log normalization**

Log normalization on scRNA-seq datasets was performed as follows: given a count matrix ($X$) where $x_{\mathrm{ij}}$ is the expression of gene i in cell j, the log-normalized version was generated, such that $y_{ij}= \log\left( \frac{x_{ij}}{\sum_{j} x_{ij}}\times{10}^{4} \right)$, where $y_{ij}$ normalized values for every gene i in every cell j⁠, respectively.

**Dimensionality reduction**

Dimensionality reduction was performed on the Alzheimer’s Disease dataset from Grubman et al(43). All dimensionality reductions (count- and binary-based) were performed with the same set of highly variable genes. These genes were identified using M3drop(47). The count-based dimensionality reduction (Principal Component Analysis, PCA) was performed using the default Seurat(48) pipeline on data that was log-normalized and scaled. The default Seurat pipeline was also applied on binary data (binary-PCA), however, without the normalization step. Additionally, for the binary-based dimensionality reduction scBFA(49) was used and eigen vectors of the jaccard cell-cell similarity matrix were calculated(Jaccard Eigen Vectors, JEVs). For the comparison of UMAP plots, the first 10 components of all four dimensionality reductions were used to calculate pair-wise Euclidian distances between cells and subsequently obtain the non-linear UMAP embeddings. The resulting plots were visually inspected on whether the cells clustered together according to previous annotations. Cell type annotations were obtained from the original study.

**Batch correction**

Three brain datasets (ADData(43), M1Data(44) and MDDData(45))were used for batch correction. From the three datasets, astrocytes, endothelial cells, microglia and oligodendrocytes were extracted, and cell type labels were harmonized. Then, the three datasets were combined and count- and binary-based PCs were calculated using Seurat. These PCs (n = 10) were used as input for Harmony(50). The uncorrected and batch corrected PCs were then used as input for the UMAP and to evaluate count- and binary-based batch corrected data.

**Use of marker genes with binary data**

To evaluate the use of marker genes with binarized scRNA-seq data, we annotated the cells from the ADData(43) dataset using markers from the BRETIGEA R-package(51). From the list of marker genes, we construct a one-hot-encoded marker matrix ($M$), where the columns represent cell types and the rows genes. When, gene $g$ is a marker for cell type $c$, then, $m_{\mathrm{gc}}=1$, otherwise $m_{\mathrm{gc}}=0$. Then, we subset the dataset, such that only known marker genes remain. Next, we calculate the Pearson’s correlation between the cell type vector ($m_{c}$) and the binarized expression of the cell ($y_{j}$). For every cell $j$ we get a measure of association ($\varphi$) with every cell type $c$. Higher values indicate higher association. As such, we annotated every cell as the cell type for which $\varphi$ is the highest. The approach was evaluated by comparing the annotations with the annotation from the original study. F1-scores for every cell type were calculated and the median F1-score was reported.

**Automatic cell-type identification**

Automatic cell-type identification was performed using two existing automatic cell-type identification methods, scPred and SingleR(52, 53). Both methods were applied to all datasets for which cell type labels were available (n = 22 out of 56). Three versions were made of every dataset, (i) a log-normalized version, (ii) a binarized version, and (iii) a shuffled version. The shuffled version was made by randomly shuffling all non-zero values of the log-normalized version. Note, that all zeros remained zero. For all three data representations, 10 reference / target splits were randomly made of 75% (reference) and 25% (target) of the total number of cells. For scPred, Seurat(48) was used to scale the data (zero-mean and standard variance) and calculate the principal components (PCs), which was done on all three data representations. Of note, the binarized data representations were not normalized. SingleR requires no specific pre-processing. The predicted labels were compared with the true labels by calculating the F1-score for every cell type and taking the median of F1-scores across all cell types, using the evaluation function of caret(54). The median F1-score of all 10 runs were used to evaluate the predictions.

**scRNA-seq data simulation and differential expression analysis**

scRNA-seq data was simulated with muscat(55) using the provided dataset(56) as reference. In total, 96 settings were generated to evaluate the performance of binarized scRNA-seq data when performing differential expression analysis (DEA) on pseudo bulk data. For the percentage of differentially expressed genes we evaluated 1%, 10%, 20%, 30%, 40%, and 50%. We evaluated datasets with 1,000, 5,000, 10,000, and 50,000 cells. And, for the number of individuals per group we evaluated 5, 10, 20, and 50 individuals. Each combination of the aforementioned settings was evaluated, resulting in the 96 settings. For each setting we generated ten datasets of 500 genes, resulting in 960 simulated datasets. Pseudo bulk data was generated with the mean as aggregation function using the aggregateData function from muscat. Here, for each individual the mean expression of each gene was calculated based on all cells belonging to the respective individual. For the binarized data, the detection rate per gene was calculated as the number of cells per individual in which the gene is observed divided by the total number of cells belonging to the respective individual. The mean pseudo bulk data was normalized using the calcNormFactors function from edgeR(57) and DEA was performed using Limma Trend(58). The t-test was used for binarized data, without normalization. P-values were corrected for multiple testing using the Benjamini-Hochberg procedure. Genes were considered significantly detected at P_adj_ ≤ 0.05.

**Identification of best count distribution model**

To test whether zero-inflation in scRNA-seq data can be explained by biological heterogeneity, we reasoned that a marker gene is a prime example of biological heterogeneity: it being highly expressed in a specific cell population while virtually absent in other cells. As such, we hypothesized that if zero-inflation is primarily explained by biological heterogeneity, marker genes should be zero-inflated. Using the same reasoning, a stably expressed gene should not be zero-inflated. We tested both hypotheses on two brain datasets(10x(44)and a Smart-Seq v2(59)). We selected two cell types and performed differential expression analyses between the cell types, using BDA(60). Next, we selected the top 100 most differentially expressed genes (sorted on P_FDR_), as well as the top 100 most stably expressed genes (sorted on smallest fold changes). Using scRATE(61), we fitted four different count distribution models (a Poisson, Negative-binomial and their zero-inflated counter parts) to all 200 genes individually. Using a leave-one-out cross validation test, we selected the best count distribution model for each gene, based on the best predictive accuracy. For every gene, we then know whether it is a marker gene or stably expressed, and whether it is zero-inflated or not. With a fisher exact test, we finally evaluate the association between zero-inflated/not zero inflated with marker/stable.

**Comparison of bit-stored and normalized datasets**

For the bit-stored datasets, the binary-based datasets were stored as Boolean vectors using the bit R-package(v4.0.4). All count-based datasets were log-normalized using Seurat(48) or normalized using scTransform(62). Before the comparison of the required storage, the normalized matrices were stored as sparse matrices.

**Magnitude recovery**

To recover the magnitude of expression from binary-based data, first pairwise cell similarities were calculated using the Jaccard index (JI). Next, for every cell, the neighbourhood is determined by the closest neighbour according to the JI, and the respective cell itself. Then, for every gene in a cell, a weighted average of the binary profile is calculated based on the neighbourhood. The weight is determined by the JI and is proportional to the sum of JIs. After this, the dataset of weighted averages is log normalized, such that $y_{ij}= \log\left( \frac{x_{ij}}{\sum_{j} x_{ij}}\times{10}^{4} \right)$, where $x_{ij}$ and $y_{ij}$ are the weighted averages and normalized values for every gene i in every cell j⁠, respectively. Finally, all non-zero values that were originally zero in the binary-based data are set to zero.

**References**

1. Aztekin,C., Hiscock,T.W., Marioni,J.C., Gurdon,J.B., Simons,B.D. and Jullien,J. (2019) Identification of a regeneration organizing cell in the Xenopus tail. *Science*, **364**, 653.

2. Bach,K., Pensa,S., Grzelak,M., Hadfield,J., Adams,D.J., Marioni,J.C. and Khaled,W.T. (2017) Differentiation dynamics of mammary epithelial cells revealed by single-cell RNA sequencing. *Nat. Commun. 2017 81*, **8**, 1–11.

3. Bacher,P., Rosati,E., Esser,D., Martini,G.R., Saggau,C., Schiminsky,E., Dargvainiene,J., Schröder,I., Wieters,I., Khodamoradi,Y., *et al.* (2020) Low-Avidity CD4 + T Cell Responses to SARS-CoV-2 in Unexposed Individuals and Humans with Severe COVID-19. *Immunity*, **53**, 1258-1271.e5.

4. Baron,M., Veres,A., Wolock,S.L., Faust,A.L., Gaujoux,R., Vetere,A., Ryu,J.H., Wagner,B.K., Shen-Orr,S.S., Klein,A.M., *et al.* (2016) A Single-Cell Transcriptomic Map of the Human and Mouse Pancreas Reveals Inter- and Intra-cell Population Structure. *Cell Syst.*, **3**, 346-360.e4.

5. Buettner,F., Natarajan,K.N., Casale,F.P., Proserpio,V., Scialdone,A., Theis,F.J., Teichmann,S.A., Marioni,J.C. and Stegle,O. (2015) Computational analysis of cell-to-cell heterogeneity in single-cell RNA-sequencing data reveals hidden subpopulations of cells. *Nat. Biotechnol.*, **33**, 155–160.

6. Bunis,D.G., Bronevetsky,Y., Krow-Lucal,E., Bhakta,N.R., Kim,C.C., Nerella,S., Jones,N., Mendoza,V.F., Bryson,Y.J., Gern,J.E., *et al.* (2021) Single-Cell Mapping of Progressive Fetal-to-Adult Transition in Human Naive T Cells. *Cell Rep.*, **34**.

7. Campbell,J.N., Macosko,E.Z., Fenselau,H., Pers,T.H., Lyubetskaya,A., Tenen,D., Goldman,M., Verstegen,A.M.J., Resch,J.M., McCarroll,S.A., *et al.* (2017) A molecular census of arcuate hypothalamus and median eminence cell types. *Nat. Neurosci. 2017 203*, **20**, 484–496.

8. Chen,R., Wu,X., Jiang,L. and Zhang,Y. (2017) Single-Cell RNA-Seq Reveals Hypothalamic Cell Diversity. *Cell Rep.*, **18**, 3227–3241.

9. Darmanis,S., Sloan,S.A., Zhang,Y., Enge,M., Caneda,C., Shuer,L.M., Gephart,M.G.H., Barres,B.A. and Quake,S.R. (2015) A survey of human brain transcriptome diversity at the single cell level. *Proc. Natl. Acad. Sci. U. S. A.*, **112**, 7285–7290.

10. Ernst,C., Eling,N., Martinez-Jimenez,C.P., Marioni,J.C. and Odom,D.T. (2019) Staged developmental mapping and X chromosome transcriptional dynamics during mouse spermatogenesis. *Nat. Commun.*, **10**.

11. Fletcher,R.B., Das,D., Gadye,L., Street,K.N., Baudhuin,A., Wagner,A., Cole,M.B., Flores,Q., Choi,Y.G., Yosef,N., *et al.* (2017) Deconstructing Olfactory Stem Cell Trajectories at Single-Cell Resolution. *Cell Stem Cell*, **20**, 817-830.e8.

12. Grün,D., Muraro,M.J., Boisset,J.C., Wiebrands,K., Lyubimova,A., Dharmadhikari,G., van den Born,M., van Es,J., Jansen,E., Clevers,H., *et al.* (2016) De Novo Prediction of Stem Cell Identity using Single-Cell Transcriptome Data. *Cell Stem Cell*, **19**, 266–277.

13. Giladi,A., Paul,F., Herzog,Y., Lubling,Y., Weiner,A., Yofe,I., Jaitin,D., Cabezas-Wallscheid,N., Dress,R., Ginhoux,F., *et al.* (2018) Single-cell characterization of haematopoietic progenitors and their trajectories in homeostasis and perturbed haematopoiesis. *Nat. Cell Biol.*, **20**, 836–846.

14. He,S., Wang,L.H., Liu,Y., Li,Y.Q., Chen,H.T., Xu,J.H., Peng,W., Lin,G.W., Wei,P.P., Li,B., *et al.* (2020) Single-cell transcriptome profiling of an adult human cell atlas of 15 major organs. *Genome Biol.*, **21**.

15. Hu,P., Fabyanic,E., Kwon,D.Y., Tang,S., Zhou,Z. and Wu,H. (2017) Dissecting Cell-Type Composition and Activity-Dependent Transcriptional State in Mammalian Brains by Massively Parallel Single-Nucleus RNA-Seq. *Mol. Cell*, **68**, 1006-1015.e7.

16. Kolodziejczyk,A.A., Kim,J.K., Tsang,J.C.H., Ilicic,T., Henriksson,J., Natarajan,K.N., Tuck,A.C., Gao,X., Bühler,M., Liu,P., *et al.* (2015) Single Cell RNA-Sequencing of Pluripotent States Unlocks Modular Transcriptional Variation. *Cell Stem Cell*, **17**, 471–485.

17. Jessa,S., Blanchet-Cohen,A., Krug,B., Vladoiu,M., Coutelier,M., Faury,D., Poreau,B., De Jay,N., Hébert,S., Monlong,J., *et al.* (2019) Stalled developmental programs at the root of pediatric brain tumors. *Nat. Genet.*, **51**, 1702–1713.

18. La Manno,G., Gyllborg,D., Codeluppi,S., Nishimura,K., Salto,C., Zeisel,A., Borm,L.E., Stott,S.R.W., Toledo,E.M., Villaescusa,J.C., *et al.* (2016) Molecular Diversity of Midbrain Development in Mouse, Human, and Stem Cells. *Cell*, **167**, 566-580.e19.

19. Lawlor,N., George,J., Bolisetty,M., Kursawe,R., Sun,L., Sivakamasundari,V., Kycia,I., Robson,P. and Stitzel,M.L. (2017) Single-cell transcriptomes identify human islet cell signatures and reveal cell-type-specific expression changes in type 2 diabetes. *Genome Res.*, **27**, 208–222.

20. Ledergor,G., Weiner,A., Zada,M., Wang,S.Y., Cohen,Y.C., Gatt,M.E., Snir,N., Magen,H., Koren-Michowitz,M., Herzog-Tzarfati,K., *et al.* (2018) Single cell dissection of plasma cell heterogeneity in symptomatic and asymptomatic myeloma. *Nat. Med.*, **24**, 1867–1876.

21. Lun,A.T.L., Calero-Nieto,F.J., Haim-Vilmovsky,L., Göttgens,B. and Marioni,J.C. (2017) Assessing the reliability of spike-in normalization for analyses of single-cell RNA sequencing data. *Genome Res.*, **27**, 1795–1806.

22. Macosko,E.Z., Basu,A., Satija,R., Nemesh,J., Shekhar,K., Goldman,M., Tirosh,I., Bialas,A.R., Kamitaki,N., Martersteck,E.M., *et al.* (2015) Highly Parallel Genome-wide Expression Profiling of Individual Cells Using Nanoliter Droplets. *Cell*, **161**, 1202–1214.

23. Mair,F., Erickson,J.R., Voillet,V., Simoni,Y., Bi,T., Tyznik,A.J., Martin,J., Gottardo,R., Newell,E.W. and Prlic,M. (2020) A Targeted Multi-omic Analysis Approach Measures Protein Expression and Low-Abundance Transcripts on the Single-Cell Level. *Cell Rep.*, **31**.

24. Kotliarov,Y., Sparks,R., Martins,A.J., Mulè,M.P., Lu,Y., Goswami,M., Kardava,L., Banchereau,R., Pascual,V., Biancotto,A., *et al.* (2020) Broad immune activation underlies shared set point signatures for vaccine responsiveness in healthy individuals and disease activity in patients with lupus. *Nat. Med.*, **26**, 618–629.

25. Marques,S., Zeisel,A., Codeluppi,S., Van Bruggen,D., Falcão,A.M., Xiao,L., Li,H., Häring,M., Hochgerner,H., Romanov,R.A., *et al.* (2016) Oligodendrocyte heterogeneity in the mouse juvenile and adult central nervous system. *Science (80-. ).*, **352**, 1326–1329.

26. Messmer,T., von Meyenn,F., Savino,A., Santos,F., Mohammed,H., Lun,A.T.L., Marioni,J.C. and Reik,W. (2019) Transcriptional Heterogeneity in Naive and Primed Human Pluripotent Stem Cells at Single-Cell Resolution. *Cell Rep.*, **26**, 815-824.e4.

27. Muraro,M.J., Dharmadhikari,G., Grün,D., Groen,N., Dielen,T., Jansen,E., van Gurp,L., Engelse,M.A., Carlotti,F., de Koning,E.J.P., *et al.* (2016) A Single-Cell Transcriptome Atlas of the Human Pancreas. *Cell Syst.*, **3**, 385-394.e3.

28. Nestorowa,S., Hamey,F.K., Pijuan Sala,B., Diamanti,E., Shepherd,M., Laurenti,E., Wilson,N.K., Kent,D.G. and Göttgens,B. (2016) A single-cell resolution map of mouse hematopoietic stem and progenitor cell differentiation. *Blood*, **128**, e20–e31.

29. Paul,F., Arkin,Y., Giladi,A., Jaitin,D.A., Kenigsberg,E., Keren-Shaul,H., Winter,D., Lara-Astiaso,D., Gury,M., Weiner,A., *et al.* (2015) Transcriptional Heterogeneity and Lineage Commitment in Myeloid Progenitors. *Cell*, **163**, 1663–1677.

30. Pollen,A.A., Nowakowski,T.J., Chen,J., Retallack,H., Sandoval-Espinosa,C., Nicholas,C.R., Shuga,J., Liu,S.J., Oldham,M.C., Diaz,A., *et al.* (2015) Molecular identity of human outer radial glia during cortical development. *Cell*, **163**, 55–67.

31. Richard,A.C., Lun,A.T.L., Lau,W.W.Y., Göttgens,B., Marioni,J.C. and Griffiths,G.M. (2018) T cell cytolytic capacity is independent of initial stimulation strength. *Nat. Immunol.*, **19**, 849–858.

32. Romanov,R.A., Zeisel,A., Bakker,J., Girach,F., Hellysaz,A., Tomer,R., Alpár,A., Mulder,J., Clotman,F., Keimpema,E., *et al.* (2017) Molecular interrogation of hypothalamic organization reveals distinct dopamine neuronal subtypes. *Nat. Neurosci.*, **20**, 176–188.

33. Segerstolpe,Å., Palasantza,A., Eliasson,P., Andersson,E.M., Andréasson,A.C., Sun,X., Picelli,S., Sabirsh,A., Clausen,M., Bjursell,M.K., *et al.* (2016) Single-Cell Transcriptome Profiling of Human Pancreatic Islets in Health and Type 2 Diabetes. *Cell Metab.*, **24**, 593–607.

34. Shekhar,K., Lapan,S.W., Whitney,I.E., Tran,N.M., Macosko,E.Z., Kowalczyk,M., Adiconis,X., Levin,J.Z., Nemesh,J., Goldman,M., *et al.* (2016) Comprehensive Classification of Retinal Bipolar Neurons by Single-Cell Transcriptomics. *Cell*, **166**, 1308-1323.e30.

35. Stoeckius,M., Zheng,S., Houck-Loomis,B., Hao,S., Yeung,B.Z., Mauck,W.M., Smibert,P. and Satija,R. (2018) Cell Hashing with barcoded antibodies enables multiplexing and doublet detection for single cell genomics. *Genome Biol.*, **19**.

36. Tasic,B., Menon,V., Nguyen,T.N., Kim,T.K., Jarsky,T., Yao,Z., Levi,B., Gray,L.T., Sorensen,S.A., Dolbeare,T., *et al.* (2016) Adult mouse cortical cell taxonomy revealed by single cell transcriptomics. *Nat. Neurosci.*, **19**, 335–346.

37. Wu,H., Kirita,Y., Donnelly,E.L. and Humphreys,B.D. (2019) Advantages of single-nucleus over single-cell RNA sequencing of adult kidney: Rare cell types and novel cell states revealed in fibrosis. *J. Am. Soc. Nephrol.*, **30**, 23–32.

38. Zeisel,A., M͡oz-Manchado,A.B., Codeluppi,S., Lönnerberg,P., Manno,G. La, Juréus,A., Marques,S., Munguba,H., He,L., Betsholtz,C., *et al.* (2015) Brain structure. Cell types in the mouse cortex and hippocampus revealed by single-cell RNA-seq. *Science*, **347**, 1138–1142.

39. Zeisel,A., Hochgerner,H., Lönnerberg,P., Johnsson,A., Memic,F., van der Zwan,J., Häring,M., Braun,E., Borm,L.E., La Manno,G., *et al.* (2018) Molecular Architecture of the Mouse Nervous System. *Cell*, **174**, 999-1014.e22.

40. Zhao,J., Zhang,S., Liu,Y., He,X., Qu,M., Xu,G., Wang,H., Huang,M., Pan,J., Liu,Z., *et al.* (2020) Single-cell RNA sequencing reveals the heterogeneity of liver-resident immune cells in human. *Cell Discov.*, **6**.

41. Zhong,S., Zhang,S., Fan,X., Wu,Q., Yan,L., Dong,J., Zhang,H., Li,L., Sun,L., Pan,N., *et al.* (2018) A single-cell RNA-seq survey of the developmental landscape of the human prefrontal cortex. *Nature*, **555**, 524–528.

42. Zilionis,R., Engblom,C., Pfirschke,C., Savova,V., Zemmour,D., Saatcioglu,H.D., Krishnan,I., Maroni,G., Meyerovitz,C. V., Kerwin,C.M., *et al.* (2019) Single-Cell Transcriptomics of Human and Mouse Lung Cancers Reveals Conserved Myeloid Populations across Individuals and Species. *Immunity*, **50**, 1317-1334.e10.

43. Grubman,A., Chew,G., Ouyang,J.F., Sun,G., Choo,X.Y., McLean,C., Simmons,R.K., Buckberry,S., Vargas-Landin,D.B., Poppe,D., *et al.* (2019) A single-cell atlas of entorhinal cortex from individuals with Alzheimer’s disease reveals cell-type-specific gene expression regulation. *Nat. Neurosci.*, **22**, 2087–2097.

44. Bakken,T.E., Jorstad,N.L., Hu,Q., Lake,B.B., Tian,W., Kalmbach,B.E., Crow,M., Hodge,R.D., Krienen,F.M., Sorensen,S.A., *et al.* (2021) Comparative cellular analysis of motor cortex in human, marmoset and mouse. *Nat. 2021 5987879*, **598**, 111–119.

45. Nagy,C., Maitra,M., Tanti,A., Suderman,M., Théroux,J.F., Davoli,M.A., Perlman,K., Yerko,V., Wang,Y.C., Tripathy,S.J., *et al.* (2020) Single-nucleus transcriptomics of the prefrontal cortex in major depressive disorder implicates oligodendrocyte precursor cells and excitatory neurons. *Nat. Neurosci.*, **23**, 771–781.

46. Senabouth,A., Andersen,S., Shi,Q., Shi,L., Jiang,F., Zhang,W., Wing,K., Daniszewski,M., Lukowski,S.W., Hung,S.S.C., *et al.* (2020) Comparative performance of the BGI and Illumina sequencing technology for single-cell RNA-sequencing. *NAR Genomics Bioinforma.*, **2**.

47. Andrews,T.S., Hemberg,M. and Birol,I. (2019) M3Drop: Dropout-based feature selection for scRNASeq. *Bioinformatics*, **35**, 2865–2867.

48. Hao,Y., Hao,S., Andersen-Nissen,E., Mauck,W.M., Zheng,S., Butler,A., Lee,M.J., Wilk,A.J., Darby,C., Zager,M., *et al.* (2021) Integrated analysis of multimodal single-cell data. *Cell*, **184**, 3573-3587.e29.

49. Li,R. and Quon,G. (2019) ScBFA: Modeling detection patterns to mitigate technical noise in large-scale single-cell genomics data. *Genome Biol.*, **20**, 1–20.

50. Korsunsky,I., Millard,N., Fan,J., Slowikowski,K., Zhang,F., Wei,K., Baglaenko,Y., Brenner,M., Loh,P. ru and Raychaudhuri,S. (2019) Fast, sensitive and accurate integration of single-cell data with Harmony. *Nat. Methods 2019 1612*, **16**, 1289–1296.

51. McKenzie,A.T., Wang,M., Hauberg,M.E., Fullard,J.F., Kozlenkov,A., Keenan,A., Hurd,Y.L., Dracheva,S., Casaccia,P., Roussos,P., *et al.* (2018) Brain Cell Type Specific Gene Expression and Co-expression Network Architectures. *Sci. Reports 2018 81*, **8**, 1–19.

52. Alquicira-Hernandez,J., Sathe,A., Ji,H.P., Nguyen,Q. and Powell,J.E. (2019) ScPred: Accurate supervised method for cell-type classification from single-cell RNA-seq data. *Genome Biol.*, **20**, 1–17.

53. Aran,D., Looney,A.P., Liu,L., Wu,E., Fong,V., Hsu,A., Chak,S., Naikawadi,R.P., Wolters,P.J., Abate,A.R., *et al.* (2019) Reference-based analysis of lung single-cell sequencing reveals a transitional profibrotic macrophage. *Nat. Immunol. 2019 202*, **20**, 163–172.

54. Kuhn,M. (2008) Building Predictive Models in R Using the caret Package. *J. Stat. Softw.*, **28**, 1–26.

55. Crowell,H.L., Soneson,C., Germain,P.L., Calini,D., Collin,L., Raposo,C., Malhotra,D. and Robinson,M.D. (2020) muscat detects subpopulation-specific state transitions from multi-sample multi-condition single-cell transcriptomics data. *Nat. Commun.*, **11**, 1–12.

56. Kang,H.M., Subramaniam,M., Targ,S., Nguyen,M., Maliskova,L., McCarthy,E., Wan,E., Wong,S., Byrnes,L., Lanata,C.M., *et al.* (2018) Multiplexed droplet single-cell RNA-sequencing using natural genetic variation. *Nat. Biotechnol.*, **36**, 89–94.

57. Robinson,M.D., McCarthy,D.J. and Smyth,G.K. (2010) edgeR: a Bioconductor package for differential expression analysis of digital gene expression data. *Bioinformatics*, **26**, 139–140.

58. Ritchie,M.E., Phipson,B., Wu,D., Hu,Y., Law,C.W., Shi,W. and Smyth,G.K. (2015) Limma powers differential expression analyses for RNA-sequencing and microarray studies. *Nucleic Acids Res.*, **43**, e47.

59. Hodge,R.D., Bakken,T.E., Miller,J.A., Smith,K.A., Barkan,E.R., Graybuck,L.T., Close,J.L., Long,B., Johansen,N., Penn,O., *et al.* (2019) Conserved cell types with divergent features in human versus mouse cortex. *Nature*, **573**, 61–68.

60. Bouland,G.A., Mahfouz,A. and Reinders,M.J.T. (2021) Differential analysis of binarized single-cell RNA sequencing data captures biological variation. *NAR Genomics Bioinforma.*, **3**.

61. Choi,K., Chen,Y., Skelly,D.A. and Churchill,G.A. (2020) Bayesian model selection reveals biological origins of zero inflation in single-cell transcriptomics. *Genome Biol.*, **21**, 183.

62. Hafemeister,C. and Satija,R. (2019) Normalization and variance stabilization of single-cell RNA-seq data using regularized negative binomial regression. *Genome Biol. 2019 201*, **20**, 1–15.
