# Supplementary material for: Consequences and opportunities arising due to sparser single-cell RNA-seq datasets
Source: Genome Biol. 2023 Apr 21;24:86. doi: 10.1186/s13059-023-02933-w (PMC10120229; doi:10.1186/s13059-023-02933-w)
Supplement: Supplementary file 3 — Additional file 3. Review history. [file 13059_2023_2933_MOESM3_ESM.docx]

Review History

**First round of review**

**Reviewer 1**

**Were you able to assess all statistics in the manuscript, including the appropriateness of statistical tests used?**

Yes, and I have assessed the statistics in my report.

**Comments to author:**

In this paper the authors demonstrated that downstream analyses based on binarized gene expressions give similar results to analyses based on count-based expressions, when the sparsity of RNA-seq data is very high.

Main concerns:
1. How does the binarization impact the visualization of cells, i.e., PCA, tSNE, or UMAP plot?
2. Definition of "detection rate" or formula for calculation?
3. The "r" in the plots of Figure 2 is confusing as the X-axis is about the Pearson correlation already.
4. Figure 3 only shows the result of 3 datasets - how about the overall results of all 52 datasets mentioned?
5. Why did the authors use the Pearson correlation sometime? And sometime used Spearman correlation?
6. Figure 3 e) p=0.99 is meaningless as the plot shows an obvious curvature.
7. Simulation in figure 3f) should be more comprehensive, i.e., check various levels of differential expression, not only 25%. How about the impact of the sparsity in the simulation data on the F1 score?
8. Under what situation (e.g., % of zeros or a specific sequence technology) the binarization results will not be acceptable?
9. How would the heterogeneousness of no-zero expressions affect the results?

**Reviewer 2**

**Were you able to assess all statistics in the manuscript, including the appropriateness of statistical tests used?**

Yes, and I have assessed the statistics in my report.

**Comments to author:**

Bouland et al. present a compelling proposal for the analysis of single cell RNA-seq datasets: represent the data as binary, such that you can store the matrices as binary (massive reduction in size, up to 17x) while still offering "similar" results to the full count matrices (the exact definition of "similar" to be still scrutinised). Overall, this is a well-organized and well-written report. I feel the results are still fairly preliminary and maybe could be expanded on to be more comprehensive, but nonetheless the results are quite intriguing and this is a discussion worth having. I have a number of comments that I think would improve the manuscript:

1. I was constantly puzzled by the result that "datasets have become sparser" argument, because that has not been my experience. I mostly analyse 10x/droplet based scRNA-seq data and maybe it's just an illusion, but the datasets seems to becoming less sparse, e.g., over the v1, v2, v3 chemistries of 10x Chromium, we get overall more UMIs per cell and more genes >0 and so I'm puzzled by Figure 1. So, there are a couple issues. For one thing, Figure 1 includes a bunch of different technologies and panel B maybe shows more that droplet tech is sparser than plate-based, than really that things are sparser over time (the X-axis is not time here). In addition, "detection rate" is not defined anywhere (that I could see), although I assume it to be something like the average per-cell percentage of genes with >0 expression. I would also suggest to simplify the figure and maybe focus on a dominant (or a few dominant) technologies. Putting them altogether when they are fundamentally different and then fitting a single curve to it is just strange/uninformative.

2. I find the whole discussion about dropouts / sparsity puzzling. Or maybe I have a too simplistic view of the situation. Basically, with droplet/10x tech, there is no dropout; you have sparsity but no dropouts. If you have multi-modal distributions, then you are mixing cells from multiple populations, but this has nothing to do with dropout, it means you should fit a mixture distribution, not a NB/Poisson to a multi-modal distribution. I find the whole discussion, which equates zeros to dropout very puzzling and I suggest that the authors present a clearer picture.

3. In the intro, they cite a paper scBFA and "improved cell type classification" and then self-cite their own work on binarised data about "improved DE analysis" as a bit of foreshadowing of what is to come in the current manuscript. I think here would be a good opportunity to spell out the *evidence* that this is the case (i.e., that you can do as-good/better with binary data), not just the citation. In order for a reader to believe that any of this is true, I would pull out the hard evidence and discuss it here. Otherwise, it feels a bit like badgering the witness.

4. The authors comment "larger number of cells has statistical benefits"; overall, I think that it not quite as simple as this. It is certainly true for some tasks (e.g., differential abundance analysis or quantifying cell types), but sparsity is the key consideration for state-level analyses. If datasets are really becoming sparser, then we are doomed, because we get access to *less* biology! If datasets are really becoming sparser, then DE for changes in cell states become more difficult to achieve because we don't see the interesting genes.

5. Figure 2: can you present the colour of points as the density; there is too much overplotting here. And while the authors want to make an argument based on the strong negative correlations, it does matter where the points are most dense.

6. In the section "to test whether counts can be discarded", they already start the analyses at cell type identification and DE. What about typical upstream analyses, like dimension reduction (which is used as input to countless things: normalisation, visualisation, GRNs, clustering, batch effect estimation/correction, etc.) and so on? What is the idea here? Can you do everything on binarised data? If so, you would need to show that all steps of the typical pipelines, wouldn't you? Maybe a Discussion item.

7. Another discussion thought: how do you compare a deeply sequenced dataset to a more shallowly sequenced dataset? Do you retain the information about how many UMIs/reads are sequenced? Because you somehow need to normalize for this, as you would expect sparsity to be a function of total depth sequenced. However, in the discussion, the authors claim that Binarization "circumvents the need to normalise", which I'm not convinced about.

8. This simulation with muscat seems weird. 10 individuals, 2500 cells per individual, so huge amount of statistical power. First of all, that is really a lot of cells per individual. The real challenge in these DE analyses and where the counts probably really help you is when you have much fewer cells per individual (e.g., 10-50). The analyses here are with respect to massive subpopulations and give no insight as to how binarization might perform for rarer cell types. In addition to this, F1-scores for DE is not what you want. Or at least, a statistician wants to know whether their tests are calibrated (whether you achieve the false discovery/positive rates that you cutoff should) and if so, then what is the power of a (reasonably) calibrated test? F1 hides all this.

9. The authors present an example 5.1GB dataset that can be stored in 300MB with binarization. Can you repeat this for all datasets, so that readers can see the distribution of gains? I'm just wondering whether 17x is the cherry-picked maximum that one could expect, or whether this is consistent across all datasets.

**Reviewer 3**

**Were you able to assess all statistics in the manuscript, including the appropriateness of statistical tests used?**

There are no statistics in the manuscript.

**Comments to author:**

Review for "The rise of sparser single-cell RNAseq datasets; consequences and opportunities"

In this paper, the authors present an interesting angle on analyzing scRNA-seq data -- the binarization of scRNA-seq counts. The authors demonstrated with statistical analysis and practical scRNA-seq applications on the impact of binarization and its relationship with the count data. I have a few specific questions:

1. In Fig 2, the authors compared three values, "log-normalized count", "binarized profile" and "detection rate". Can the author define "detection rate" with math notations and state the mathematical relationship between "log-normalized count" and "binarized profile"? With the notations, corr(log-normalized count, binarized profile) can be computed, which is a function of non-zero entries in the "log-normalized count". Thus, it's not surprising that "sparsity" is correlated with this correlation. This is a general phenomenon for any binarization, not specific to scRNA-seq data. Through the formulation of this correlation, please comment on how the number of cells, the number of genes, sparsity and detection rate affect binarization.

2. How are missing values accounted for in such binarization process? How does the handling of missing values in binarization affect the downstream analyses?

3. In the evaluation, the authors presented supervised cell type classification methods. How does binarization affect unsupervised learning? Can the authors present such results using unsupervised methods, such as Seurat and Harmony?

4. Integration has been an intriguing task in scRNA-seq data analysis. How does the binarization of gene expression counts affect scRNA-seq integration?

5. The gene expression analysis used a simulated dataset. Can the authors present the top differentially expressed genes identified in a real dataset with multiple cell types (with and without binarization)? Can the binarized data identify all the top differential genes?

6. In Figure 3c and 3d, the authors presented the classification result using SingleR. Can the authors comment on why there is a big drop in F1-score and accuracy for the shuffled datasets?

7. How does binarization affect the runtime and memory usage of the downstream analysis tasks presented in this work?

**Reviewer 4**

**Were you able to assess all statistics in the manuscript, including the appropriateness of statistical tests used?**

Yes, and I have assessed the statistics in my report.

**Comments to author:**

Bouland et al present a thought-provoking argument that scRNA-seq data analysis practitioners should more strongly and more regularly consider binarising scRNA-seq count data and then conduct downstream analysis on binary data (0, 1) rather than on the transcript/UMI count data usually used. The authors make some compelling arguments that binarisation should be used more often and the concise data analyses are effective in supporting their arguments. The crux is, they argue, that binarisation could/would/should make common analysis tasks more computationally efficient while sacrificing little in terms of accuracy or utility of the results obtained.

However, the paper in its current form completely lacks any description of methods used, so it is impossible to evaluate the rigour and accuracy of the data analysis results presented. Furthermore, there are several aspects of the argument/discussion that in my view need to be deepened and strengthened. If done, then I think this paper could make a useful Short Report, or similar, that would serve as a useful spur for discussion in the scRNA-seq data analysis community.

An alternative would be to shorten the manuscript and present it purely as an opinion/editorial article, in which case it could productively spur discussion in the field without requiring the depth and rigour of a Short Report or "full" paper.

As the comments below will show, I have a lot of issues with the paper in its current form. Overall, those issues are more related to the framing, discussion and presentation of the argument rather than the results, which I found interesting and informative. This paper prompted me to think further about this conundrum in scRNA-seq data analysis, but there is much that could be improved to make this paper a very valuable contribution to thinking in the field.

Major Comments:
⁃ No Methods section!
⁃ What about marker genes? In my experience, the actual expression level of marker genes used to define cell types is highly informative. Binarising data from marker genes is likely to remove the signal and diminish their utility. Ideally, the authors could provide some analyses addressing the issue of removing signal through binarising data - most obviously relevant in the case of marker genes, but other scenarios must also exist. At the very least the authors should
⁃ The existence of ambient RNA ("background" RNA present from lysed cells and captured in droplets along with in-tact cells) poses substantial theoretical problems with binarisation. The authors claim that "binarization alleviate noise, as it is only subject to detection noise opposed to quantification and detection noise". However, under binarization ambient RNA will yield "detection" of a gene in a cell at the same level as true, endogenous (and possibly high) expression of a gene. In such cases, binarization will add detection noise compared to count-based quantification. This point warrants serious discussion in this paper (ideally supported by relevant analyses).
⁃ The authors claim: "standard count distribution models seem to fail in explaining the excess of zeros" (p1, l33) - this claim needs more discussion and evidentiary support. They cite a few papers supporting this view, but there are (at least as) many papers arguing the opposite - that sparse scRNA-seq count data is not zero-inflated (typically stated w.r.t. the negative binomial distribution).
⁃ Authors should avoid the term "dropouts" (p1 l39) - this is a muddied and unhelpful term. If they're talking about "observed zeros", then they should just use that term; if they're referring to other types (biological/technical) of zeros, then they should be specific. Jiang et al (2022) provide a very useful taxonomy of different types of observed zeros in scRNA-seq data - their definitions should better inform the discussion in this paper.
⁃ Overall, I find the introduction (first two paras on p1) to be superficial and lacking. The start of the Discussion is much better, with mention of the Jiang et al and Sarkar and Stephens papers. A deeper, more mathematical introduction to the problem (binarization vs counts) that engages properly with these papers would drastically improve the introduction/framing of the paper. Unfortunately, the authors' claim that "this model [the Sarkar and Stephens model] is not suited for the observed zero inflation" is not strictly true. The hierarchical model proposed uses a Poisson model for the sampling variation of counts - with appropriate parameter definition in the hierarchical data generative model, the Sarkar-Stephens model can indeed account for "zero inflation" (if this exists - again, the claim of zero inflation only makes sense w.r.t. a specific model). I'm not convinced that the authors have fully absorbed the messages of the Sarkar and Stephens paper - as such, deeper and more rigorous discussion and treatment of that model is warranted here.
⁃ The details of how scRATE are very important for assessing the accuracy of the enrichment results presented in Table 1 - without complete Methods we can't evaluate these results appropriately.
⁃ Were scPred and SingleR pre-trained on binarised counts for this application, or pre-trained on raw counts? (Again, a Methods section should remove the need for confusion for the reader on such points.)
⁃ The authors claim that binarisation has the benefit of not needing to fuss about normalization/transformation. While that may be possibly true for the cell type identification and differential expression analyses shown in this paper, it is not true for any dimension reduction analyses (among other analyses one might do). For comparison, there is currently much debate on how to best apply flavours of TF-IDF (term frequency - inverse document frequency) normalization/transformation in the analysis of binary scATAC-seq data. I'm not at all persuaded that binarization of scRNA-seq data avoids complexities or concerns about normalization/transformation for many downstream analyses.


Minor Comments:
- the abbreviations "scRNAseq" and "scRNA-seq" are both used in the abstract - choose one and be consistent
- p4 l23: "Sakar" should be "Sarkar"
- More than once, in-text citations of papers contain misspellings of authors' names ("Sakar", "Jian") - please make sure these are correct

Best wishes,
Davis J. McCarthy

**Authors Response**

**Point-by-point responses to the reviewers’ comments:**

**Response to Reviewer 1**

In this paper the authors demonstrated that downstream analyses based on binarized gene expressions give similar results to analyses based on count-based expressions, when the sparsity of RNA-seq data is very high.

Main concerns:

1. How does the binarization impact the visualization of cells, i.e., PCA, tSNE, or UMAP plot?

**Response:** In the previous version of the manuscript we cited scBFA(1), which is a dimensionality reduction method relying on binarized scRNA-seq data. This method was shown to improve visualization and classification of cell identity and trajectory inference compared to methods using counts. To emphasize this, we now more explicitly refer to this method in the introduction. However, the scBFA paper did not evaluate the effects of binarization on visualizations using tSNE or UMAP. Therefore, we performed a comparison experiment in which we used three different dimensionality reduction approaches on binarized scRNA-seq data and compared it to count-based approaches. The reduced dimensions, as well as UMAPs generated with the various approaches were compared visually and quantitatively. Visually, the UMAPs generated using binary and count data looks very similar (**Fig. 2a-b**). Overlaying cell annotations (determined by the original authors of the data based on count-based analysis) shows that cells with the same label cluster together in the UMAP regardless of whether it is generated using binary or count data. Quantitively, in UMAP space, the binarized data shows a slightly better silhouette score for the known cell types than count data. (i.e. a silhouette score of 0.44 for binarized data with respect to 0.35 for count data, **SFig. 4**). Based on these observations, we conclude that the binarization is not impacting the visualization of cells.

***Changes to the introduction***: “*For instance, scBFA(14), a dimensionality reduction method for binarized scRNA-seq data, showed improved visualization and classification of cell identity and trajectory inference when compared to methods that use count data.”*

***Changes to the results and discussion***: “*To assess whether counts can actually be discarded in practice, we assessed whether binarized data can give comparable results to counts in four common single-cell analysis tasks: (1) dimensionality reduction for visualization, (2) data integration, (3) cell type identification, and (4) differential expression analysis using pseudobulk. First, for dimensionality reduction, we used three different dimensionality reduction approaches on binarized scRNA-seq data; (i) scBFA (14), (ii) PCA (Fig. 2a), and (iii) eigenvectors of the Jaccard cell-cell similarity matrix (see Supplementary Methods). All three approaches were compared to the standard approach of applying PCA to the normalized counts (Fig. 2b, SFig. 2). Further, for all four methods the first ten components were used to generate a non-linear embedding using UMAP (SFig. 3). Qualitatively, we observed that the results of binary-based dimensionality reduction are comparable to standard count-based methods. Especially the UMAP generated with the binary-based PCs was visually very similar to the UMAP generated with the count-based PCs (Fig. 2c-d). Calculating the silhouette score (SS) for each cell type with the reduced dimensions (n = 10) resulted in slightly lower scores for scBFA (SS = 0.32) and binary-based PCA(SS = 0.39) compared to the count-based PCA (SS = 0.44) (SFig. 4). However, in the UMAP space (2-dimensional), silhouette scores for scBFA (SS = 0.43) and binary-based PCA (SS = 0.42) were higher than count-based PCA (SS = 0.35). ”*

***Changes to the*** ***Supplementary Methods***: We added a section describing the procedures for the dimensionality reductions and the procedure used to integrate the three different datasets, as well as showing the resulting UMAP and visualizations

2. Definition of "detection rate" or formula for calculation?

**Response:** We thank the reviewer for noticing this oversight. We have added the definition to the supplementary methods.

***Changes to the supplementary methods***: *“Binarized scRNA-seq datasets were generated by transforming the raw count matrix such that a zero remains a zero and every non-zero value is assigned a one. The detection rate refers to the fraction of non-zero values. More formally, binarized scRNA-seq data is generated as follows:*

$y_{ij}= \left\{ \begin{matrix} 1 & x_{ij} \geq1 \\ 0 & otherwise \end{matrix} , for i\in\left[ 1,g \right], j\in[1,n] \right.$

*where* $x_{ij}$ *is the expression of gene i in cell j; g is number of genes and n the number of cells in the dataset.*

*The detection rate* ${DR}_{j}$ *for cell j is then defined as:*

${DR}_{j}= \frac{\sum_{i=1}^{g} y_{ij}}{g}, \forall j\in[1,n]$

*Similarly, we define the detection rate for a gene* ${DR}_{i}$ *and the detection rate across the whole dataset* ${DR}_{d}$*:*

${DR}_{i}= \frac{\sum_{j=1}^{n} y_{ij}}{n}, \forall i\in[1,g]$

${DR}_{d}= \frac{\sum_{i=1}^{g} \sum_{j=1}^{n} y_{ij}}{ng}$

*Note, the detection rate of a gene can be determined either within a specific cell population or within the dataset.”*

3. The "r" in the plots of Figure 2 is confusing as the X-axis is about the Pearson correlation already.

**Response:** We apologize for the confusing notation. We have now changed the plot labels and avoid using “r”. Inspired by your comment #4, we have now extended the analysis presented in Figure 2 (Fig. 1c-d in revised manuscript) to all 56 datasets, showing that the trend observed initially in 4 datasets generalizes to all 56 datasets.

***Changes in the results and discussion***: We replaced *r* to *alfa* in the plot and changed the label of the *x*-axis to “*correlation between binary and normalized expression (p)*”. Moreover, we have added a more extensive description of the figure in the caption:

“Figure 1: *B) Scatterplot of the detection rate (y-axis) against the number of cells (log scale, x-axis). C) On the x-axis the Pearson’s correlation coefficient (p) of every cell from the PaulHSC dataset between the binarized and normalized expressions. On the y-axis the product of the detection rate and the variance of the non-zero values (q). α is the Pearson’s correlation coefficient between these values p and q across all cells. D) Boxplots of the α-values for all 56 datasets grouped by technology. One dataset (LawlorPancreasData) was excluded as α-value (α = 0.42) for this dataset was a clear outlier.”*

4. Figure 3 only shows the result of 3 datasets - how about the overall results of all 52 datasets mentioned?

**Response:** Initially we restricted ourselves to these three datasets as cell type labels were readily available. We have now extended the analysis to 22 (out of the 56 datasets), for which we eventually could acquire cell type labels. Additionally, to have a better understanding of the variability of the results, we ran ten different reference/target splits for each dataset and report the median F1 score across these ten splits. Overall, our original conclusion remains valid, i.e. for the task of automatic cell type prediction binarized data performs similarly to normalized count data.

***Changes in the results and discussion****: “Next, we compared the performance of automatic cell type identification using scPred and SingleR(25, 26) on 22 datasets for which cell type annotations were available. The median F1-scores were highly similar between cell type identifications based on the binarized and the normalized count data, despite large variation of sparseness between these datasets. This finding implies that counts do not add information for cell type identification. This conclusion was further supported by randomly shuffling the non-zero counts, which resulted in a comparable performance (Fig. 2I, SFig. 9).*

5. Why did the authors use the Pearson correlation sometime? And sometime used Spearman correlation?

**Response:** We used the Spearman correlation only when we assessed the association between the mean expression and the detection rate of pseudobulk aggregated data. We did so because this relationship is known to be non-linear as reported earlier (4, 5) (see also SFig. 10), but their ranks are linearly correlated. For all other analyses, we tested for a linear relationship and thus used the Pearson correlation. We have added our justification in the methods.

6. Figure 3 e) p=0.99 is meaningless as the plot shows an obvious curvature.

**Response:** The reported value (p=0.99) is the Spearman rank correlation which does not test whether the values are correlated but whether the rank positions are correlated (see also answer at comment 5).

7. Simulation in figure 3f) should be more comprehensive, i.e., check various levels of differential expression, not only 25%. How about the impact of the sparsity in the simulation data on the F1 score?

**Response:** We thank the reviewer for this suggestion. We have extended the simulation experiment to test six different levels of differential expression (1%, 10%, 20%, 30%, 40%, and 50%), four different sample sizes (5 vs 5, 10 vs 10, 20 vs 20, and 50 vs 50) and four different dataset sizes (1000, 5000, 10000, and 50000 cells), resulting in 96 unique simulation settings. Additionally, each combination of parameters was ran 10 times to assess the variability of the results.

In addition, we have also looked at the impact of the sparsity on the differential expression analyses. We found that highly expressed genes (low sparsity, high detection rate ≥ 0.9) are more difficult to identify as differentially expressed using the binarized representation, especially with low number of samples and cells. However, with increasing sample sizes and number of cells, the binarized representation starts to perform similarly and eventually even outperforms count based representation in this simulation setting.

***Changes to the results and discussion***: “*To quantify this further, we generated 960 datasets using muscat (18) with 96 unique simulation settings (see Supplementary Methods). In each dataset, pseudobulk data for each individual was generated and we identified differential expressed genes using Limma trend(28) for the mean gene expression and a t-test for the detection rate. In general, the F1-scores for the count and binary representations were very similar across the different settings, however, with small sample sizes and fewer cells, analyses based on a count representation performed better, while analyses based on a binarized expression performed better with larger sample sizes and more cells (SFig. 11). Additionally, count-based analyses resulted in more false positives (SFig. 12) while binarized-based analyses resulted in more false negatives (SFig. 13). The false negatives were primarily due to highly expressed genes that show no differences in the detection rate. At larger sample sizes and with more cells, the false negatives diminished (SFig. 14). All together, these result show that most of the information is indeed captured in the binary representation, only when genes have a high detection rate (>0.9), then, changes in expression are not reflected in the binary representation and, consequently, information from counts is needed.”*

8. Under what situation (e.g., % of zeros or a specific sequence technology) the binarization results will not be acceptable?

**Response:** Following your previous comment, we expanded the simulation experiment with testing varying degrees of sparseness (see also answer to comment #7). We find that for genes with a detection rate ≥ 0.90, a binarized representation shows excessive false negatives. This is in agreement with previous findings by Li. R et al(1) who reported that for the task of dimensionality reduction binarization results are not acceptable anymore for a detection rate of ≥0.9. Besides changes mentioned in answer to comment #7, we added a remark on this finding in the discussion.

***Changes in the results and discussion***: *“Previous work suggested that when the detection rate is >90%, visualizations based on the binary representation do not perform on par with count-based representation (14). With our simulation experiments, we have shown a similar trend when considering the task of detecting differential expressed genes based on pseudobulk values.”*

9. How would the heterogeneousness of no-zero expressions affect the results?

**Response:** In the previous version of the manuscript, we showed that the detection rate of a cell correlates with the strength of the correlation between the binarized and normalized representations (***SFig. 1a***). When incorporating the variance of the non-zero counts of the normalized profile (***SFig. 1b***), this correlation improves even further. This suggests that more signal is lost with the binarized representation when there is a high variance (heterogeneousness) of the non-zero expression values. However, we extended the automatic cell type prediction analysis to 22 datasets. And, despite all the varying degrees of variance in the datasets, the binarized representations remained performing similarly to counts. As such, we believe that the heterogeneousness of the non-zero expression have little effect on the results.

***Changes in the results and discussion***: “*This strong correlation is primarily explained by the detection rate (SFig. 1a) and the variance of the non-zero counts of a cell (SFig. 1b). In cells where the detection rate is low (many zeros) and the variance of the non-zero counts is small, the correlation between the normalized expression values and their binary representation is high (Fig. 1c). Across all datasets, the detection rate and variance of measured expressions were good predictors for the correlation between the binary representation and the normalized representation, although differences between technologies exist (Fig. 1d).”*

***Changes in the results and discussion***: “*The median F1-scores were highly similar between cell type identifications based on the binarized and the normalized count data, despite large variation of sparseness between these datasets.”*

**Response to Reviewer 2**

Bouland et al. present a compelling proposal for the analysis of single cell RNA-seq datasets: represent the data as binary, such that you can store the matrices as binary (massive reduction in size, up to 17x) while still offering "similar" results to the full count matrices (the exact definition of "similar" to be still scrutinised). Overall, this is a well-organized and well-written report. I feel the results are still fairly preliminary and maybe could be expanded on to be more comprehensive, but nonetheless the results are quite intriguing and this is a discussion worth having. I have a number of comments that I think would improve the manuscript:

1. I was constantly puzzled by the result that "datasets have become sparser" argument, because that has not been my experience. I mostly analyse 10x/droplet based scRNA-seq data and maybe it's just an illusion, but the datasets seems to becoming less sparse, e.g., over the v1, v2, v3 chemistries of 10x Chromium, we get overall more UMIs per cell and more genes >0 and so I'm puzzled by Figure 1. So, there are a couple issues. For one thing, Figure 1 includes a bunch of different technologies and panel B maybe shows more that droplet tech is sparser than plate-based, than really that things are sparser over time (the X-axis is not time here). In addition, "detection rate" is not defined anywhere (that I could see), although I assume it to be something like the average per-cell percentage of genes with >0 expression. I would also suggest to simplify the figure and maybe focus on a dominant (or a few dominant) technologies. Putting them altogether when they are fundamentally different and then fitting a single curve to it is just strange/uninformative.

**Response:** First, we apologize for not properly defining the detection rate. This remark was also raised by Reviewer #1 and we refer to our answer of her/his Comment #2. Continuing, we indeed agree that with improved chemistries (e.g., 10x v3 vs 10x v2), more UMIs are detected. However, although with every iteration of the 10x chemistries more UMIs can be detected, there is also a tradeoff between sequencing depth (number of UMIs per cell) and total number of cells sequenced. Hence, the trend that we observe is most likely influenced by prioritizing the number of cells sequenced over the number of UMIs per cell. We understand the argument regarding the selection of one dominant technology to show in Figure 1. However, we would like to show that, regardless of the technology used, the resulting gene by cell matrix that is used as input for downstream tasks are getting sparser (more zero measurements) over time. And it is the increased sparsity that complicates downstream tasks. Nevertheless, we have added a remark on the notion of the improved chemistry.

***Changes to the results and discussion***: “*Another clear trend that can be seen is that an increasing number of cells is highly correlated with decreasing detection rates (fraction of non-zero values) (Pearson’s correlation coefficient of r = -0.47, Fig. 1b). Note that this trend of measuring more cells per dataset outweighs improved chemistry over time, and thus still results in sparser datasets”*

2. I find the whole discussion about dropouts / sparsity puzzling. Or maybe I have a too simplistic view of the situation. Basically, with droplet/10x tech, there is no dropout; you have sparsity but no dropouts. If you have multi-modal distributions, then you are mixing cells from multiple populations, but this has nothing to do with dropout, it means you should fit a mixture distribution, not a NB/Poisson to a multi-modal distribution. I find the whole discussion, which equates zeros to dropout very puzzling and I suggest that the authors present a clearer picture.

**Response:** We completely agree with the reviewer on these remarks. Due to inconsistent use of the term “dropout” in literature, we deliberately avoid using the term in the manuscript (mentioned only once when referring to the paper by Qui. P(6), since they used the term “dropout” for all zeros). We agree that, in hindsight, our initial discussion was too much spread out over the introduction and discussion so that our message was somewhat lost. Therefore, we now moved the discussion of zero-inflation and zeros to the introduction and rewrote it to make the point clearer. Briefly, for binarization, it is important that the detection pattern captures biological information. Referring to Sarkar and Stephens, we explain why we believe this is true: 1) a transcript can be truly absent, hence it encodes useful information; or 2) you failed to measure a transcript, hence, it is unlikely to be highly expressed. Consequently, all zeros encode useful information.

**Changes in the introduction: “***Jiang et al.(8) discuss the ‘zero-inflation controversy’, in which a distinction is made between a biological zero, indicating the true absence of a transcript, and a non-biological zero, indicating failure of measuring a transcript that was present in the cell. Similarly, Sarkar and Stephens(11) make a distinction between measurement and expression. They proposed a model that is a combination of an expression model that encodes the true absence of a transcript, i.e. a (biological) zero, with a measurement model, for which they use a Poisson model (which can result in non-biological zeros due to limited sequencing depth). Consequently, even non-biological zeros encode useful biological information as then the gene is unlikely to be highly expressed. Or, in other words: all zeros in scRNA-seq datasets have biological significance.”*

3. In the intro, they cite a paper scBFA and "improved cell type classification" and then self-cite their own work on binarised data about "improved DE analysis" as a bit of foreshadowing of what is to come in the current manuscript. I think here would be a good opportunity to spell out the *evidence* that this is the case (i.e., that you can do as-good/better with binary data), not just the citation. In order for a reader to believe that any of this is true, I would pull out the hard evidence and discuss it here. Otherwise, it feels a bit like badgering the witness.

**Response:** We thank the reviewer for this comment, and we now elaborate more about the cited methods.

**Changes in the introduction:** “*For instance, scBFA(14), a dimensionality reduction method for binarized scRNA-seq data, showed improved visualization and classification of cell identity and trajectory inference when compared to methods that use count data. Likewise, we introduced Binary Differential Analysis (BDA)(15), a differential expression analysis method relying on binarized scRNA-seq data. We showed that differential expression analysis on binary representations of scRNA-seq data faithfully captures biological variation across cell types and conditions. As scRNA-seq datasets are becoming increasingly bigger and sparser, we wondered if binary should be the preferred data representation for other tasks. In this work, we explore the consequences of sparser datasets and the applicability of binarized scRNA-seq data for various single-cell analysis tasks.*”

4. The authors comment "larger number of cells has statistical benefits"; overall, I think that it not quite as simple as this. It is certainly true for some tasks (e.g., differential abundance analysis or quantifying cell types), but sparsity is the key consideration for state-level analyses. If datasets are really becoming sparser, then we are doomed, because we get access to *less* biology! If datasets are really becoming sparser, then DE for changes in cell states become more difficult to achieve because we don't see the interesting genes.

**Response:** We agree that this statement was too simplistic. However, it is also not as simple as that sparser datasets result in less biology. Certainly not when the increased sparsity is partially due to prioritizing measuring more cells over sequencing depth. It even has been shown that measuring more cells increases the expression probability and overall detection power more than deeper sequencing does(7). Also, most single-cell analyses are at a cell type level, so one considers an aggregate of cells with which the sparsity is countered. We removed our initial simplistic statement and provided a bit more nuance on this discussion in the paper.

**Changes in the *results and discussion*:** “*It is likely that this trend will continue over the next years as shallow sequencing of many cells is more cost effective than deep sequencing of a few cells(16). Moreover, measuring more cells increases the overall power to detect genes in a given dataset (17). This trend will be amplified, as more population scale and multi-condition scRNA-seq datasets are emerging (17, 18), for which a low coverage sequencing is sufficient to capture cell type specific gene expression (given enough cells are measured per individual and per cell type)(19). Altogether, these developments will result in sparser scRNA-seq datasets with larger numbers of cells.”*

5. Figure 2: can you present the colour of points as the density; there is too much overplotting here. And while the authors want to make an argument based on the strong negative correlations, it does matter where the points are most dense.

**Response:** We thank the reviewer for this suggestion. We have now changed Figure 2 to incorporate the variance of non-zero counts, explaining the correlation between the binarized representation and count-based representation better (in response to Comments 3 and 9 of Reviewer 1). All data points (cells) are now densely packed around the linear model line. Consequently, the density of the points does not add additional information.

6. In the section "to test whether counts can be discarded", they already start the analyses at cell type identification and DE. What about typical upstream analyses, like dimension reduction (which is used as input to countless things: normalisation, visualisation, GRNs, clustering, batch effect estimation/correction, etc.) and so on? What is the idea here? Can you do everything on binarised data? If so, you would need to show that all steps of the typical pipelines, wouldn't you? Maybe a Discussion item.

**Response:** In the introduction section, we already highlighted various methods using binarized data for some of the tasks mentioned by the Reviewer, including: 1) dimensionality reduction scBFA(1); 2) gene regulatory network inference(8), 3) clustering (6); and 4) differential expression analysis(9). Motivated by the Reviewer’s comment, we have now added additional experiments in which we show that binarized data can be used for dimensionality reduction and data integration as well using commonly used pipelines.

We applied the “Seurat pipeline” (excluding normalization) on binarized data and compared the resulting UMAPs with UMAPs based on normalized counts. In addition, we performed batch correction with Harmony when integrating three different datasets and compared the resulting integrations based on binarized vs count data. Overall, the results show comparable results for the binarized representation and the count-based representations. Altogether, our result, together with existing literature, show that binarized data is indeed sufficient for many single-cell analyses tasks like: dimensionality reduction, visualization, clustering, trajectory inference, batch correction, differential expression analysis, cell type prediction and generating pseudo-bulk data.

**Changes in *results and discussion*:** “*Second, we integrated three scRNA-seq datasets(20–22) with Harmony(23), using count- and binary-based PCA. Both, visually and quantitatively, we observed an improved mixing of cells for the binary representation (LISI = 1.18) as compared to counts (LISI = 1.12) (SFig. 5-6).”*

**Changes in the conclusion:** “*Concluding, our results support existing literature in showing that binarized scRNA-seq data can be used for: dimensionality reduction, data integration, visualization, clustering, trajectory inference, batch correction, differential expression analysis and cell type prediction. We believe scRNA-seq tool developers should be aware of the possibility of using a binary representation of the scRNA-seq data instead of count-based data, as it gives opportunities to develop computational- and time-efficient tools.*”

7. Another discussion thought: how do you compare a deeply sequenced dataset to a more shallowly sequenced dataset? Do you retain the information about how many UMIs/reads are sequenced? Because you somehow need to normalize for this, as you would expect sparsity to be a function of total depth sequenced. However, in the discussion, the authors claim that Binarization "circumvents the need to normalise", which I'm not convinced about.

**Response:** We agree that the sequencing depth (number of reads per cell) influences the detection rate. To quantify this effect, we have added a simulation over different detection rates (also as a reaction to Comment 7 and 8 of Reviewer 1). Indeed, when the detection rate becomes high (>0.9) binarizing data can worsen performance on downstream tasks. However, in case enough cells are measured, the effects seem to disappear for lower detection rates. For a more elaborate discussion on this we refer to answers to the referred comments of Reviewer 1.

Although we agree in principle that the sequencing depth indeed should be taken into account (for example when merging datasets having different sequencing depths), we observed on-par performance of all tested downstream tasks based on the binarized representation as compared to those based on the count-based representation. Please note, that no normalization was done for the binary data, whereas we did normalize the count-based data. Given those results, we still believe that the influence of sequencing depth is much more moderate for the binary representation in comparison to the count-based representation.

Nevertheless, we now have removed the claim that “normalization can be circumvented” and instead have a more toned-down discussion.

**Changes to the *results and discussion*:** “*For the various tasks that we explored, such as dimensionality reduction, data integration, cell type prediction, differential expression analysis(15) and clustering(12), the binary representations required no normalization. Hence various subjective choices on the normalization could be avoided, which improves reproducibility of these tasks. However, as sequencing depth has an effect on the detection rate of a cell, it is likely this is not the case for all downstream tasks.*”

8. This simulation with muscat seems weird. 10 individuals, 2500 cells per individual, so huge amount of statistical power. First of all, that is really a lot of cells per individual. The real challenge in these DE analyses and where the counts probably really help you is when you have much fewer cells per individual (e.g., 10-50). The analyses here are with respect to massive subpopulations and give no insight as to how binarization might perform for rarer cell types. In addition to this, F1-scores for DE is not what you want. Or at least, a statistician wants to know whether their tests are calibrated (whether you achieve the false discovery/positive rates that you cutoff should) and if so, then what is the power of a (reasonably) calibrated test? F1 hides all this.

**Response:** We thank the reviewer for this comment. Reviewer 1 had a very similar comment. Both suggestions prompted us to setup a more extensive simulation experiment which covers a wider range of parameter settings. For a more detailed answer we like to refer to our response to comment 7 of Reviewer 1. We would like to argue that 2500 cells per individual is not uncommon nowadays, especially with the emergence of population scale and multi-condition scRNA-seq datasets(10).

9. The authors present an example 5.1GB dataset that can be stored in 300MB with binarization. Can you repeat this for all datasets, so that readers can see the distribution of gains? I'm just wondering whether 17x is the cherry-picked maximum that one could expect, or whether this is consistent across all datasets.

**Response:** We performed this analysis on all 56 datasets. Initially, we only compared the binarized representation with data normalized using scTransform. We have now added a comparison with log-normalized data as well. Across all datasets, we found an 11-fold reduction of storage when compared to scTranform, and a 4-fold reduction when compared to log-normalized data. We changed the text in the manuscript accordingly and added a new figure to the supplements.

**Changes to the *results and discussion*: “***Increasingly larger datasets require increasingly more computational resources. The storage required for all 56 datasets used in this study was 764 Gigabytes after normalization using sctransform(30), or 276 Gigabytes when log-normalized and stored as sparse matrices. In contrast, binarizing the same datasets and storing them as bits required only 73 Gigabytes, which is an ~11-fold and ~4-fold reduction in storage requirements, respectively (SFig. 15). Yet, there are big differences across datasets. For example, a reduction of ~50-fold and ~20-fold, respectively was acquired for the BuettnerESC dataset(31). The amount of storage that can be saved is highly correlated with the detection rate (SFig. 16), with the highest gain for datasets with a high detection rate. The considerable storage reduction of the binary representation gives the potential to boost downstream analyses to larger numbers of cells, opening possibilities to get a more fine-grained resolution of biological heterogeneity(32).*

**Changes in supplement section:** We added figures SFig. 15 and SFig. 16 to the supplement.

**REVIEWER 3**

Reviewer #3: Review for "The rise of sparser single-cell RNA-seq datasets; consequences and opportunities"

In this paper, the authors present an interesting angle on analyzing scRNA-seq data -- the binarization of scRNA-seq counts. The authors demonstrated with statistical analysis and practical scRNA-seq applications on the impact of binarization and its relationship with the count data. I have a few specific questions:

1. In Fig 2, the authors compared three values, "log-normalized count", "binarized profile" and "detection rate". Can the author define "detection rate" with math notations and state the mathematical relationship between "log-normalized count" and "binarized profile"? With the notations, corr(log-normalized count, binarized profile) can be computed, which is a function of non-zero entries in the "log-normalized count". Thus, it's not surprising that "sparsity" is correlated with this correlation. This is a general phenomenon for any binarization, not specific to scRNA-seq data. Through the formulation of this correlation, please comment on how the number of cells, the number of genes, sparsity and detection rate affect binarization.

**Response:** We apologize for the missing notation. The detection rate is the fraction of non-zeros. This can be calculated per gene, i.e. the fraction of non-zero expression measurements across all cells; or per cell, i.e. the fraction of non-zero expressions across all genes. With the binarized profile we refer to a binary representation of all genes within one cell (vector of zero and ones). Reviewer 1 had a similar remark, and we further refer to Comment #2 of Reviewer #1 for a detailed description of the detection rate.

We agree with the reviewer that the correlation of the binary profile and the normalized count profile is largely determined by the number of zeros in the normalized data. Yet, this relationship is also partially determined by the variance of the non-zero counts. This prompted us to look at the combination of this variance with the detection rate when considering the correlation between the detection rate and the normalized transform. Since a similar remark was raised by Reviewer 1, we refer to our response to Comment 9 of Reviewer 1.

2. How are missing values accounted for in such binarization process? How does the handling of missing values in binarization affect the downstream analyses?

**Response:** We purposefully do not make any distinction between different types of zeros. In the introduction we now reason why the detection pattern captures biological information. Briefly, a transcript can be truly absent; hence, it encodes useful information. Or, we failed to measure a transcript; hence, it is unlikely to be highly expressed. Consequently, all zeros encode useful information. For a further response we refer to comment 2 of Reviewer 2 who also addressed the discussion around zeros.

3. In the evaluation, the authors presented supervised cell type classification methods. How does binarization affect unsupervised learning? Can the authors present such results using unsupervised methods, such as Seurat and Harmony?

**Response:** We would like to refer to Comment 6 of reviewer 2 which addresses this point. Briefly, we have now run the Seurat pipeline on three scRNA-seq datasets, both binarized and counts, to generate UMAP plots and show that the different data representations show no visual improvement over the other. Evaluation of the clustering of binarized data was previously evaluated by Peng Qui(6), which we refer to in the intro.

4. Integration has been an intriguing task in scRNA-seq data analysis. How does the binarization of gene expression counts affect scRNA-seq integration?

**Response:** Reviewer 2 raised the same question, which we addressed by adding new experiments to evaluate integration using binarized data. Results show that similar data integration can be achieved for binarized data in comparison to normalized count-based data. We refer to the answer to Comment 6 from Reviewer 2 for more details.

5. The gene expression analysis used a simulated dataset. Can the authors present the top differentially expressed genes identified in a real dataset with multiple cell types (with and without binarization)? Can the binarized data identify all the top differential genes?

**Response:** For a full discussion on this comment, we refer to our previously published work in which we presented binary differential analysis (BDA)(9). Briefly, BDA relies on the differences in proportion of zeros and ones across groups (cell types or conditions). We compared BDA with count-based methods and found a very high degree of agreement between binary-based and count-based methods, also for the top differential genes. Moreover, the log odds ratio of the binary test has a strong correlation with log fold change. We included the below figure showing these results (**R Fig 1**).

We would like to note that in reaction to Comment 7 of Reviewer 1, we extended the simulation analysis considerably. As a result, we found that with small sample sizes and fewer cells the binary representation performs worse than the count-based representation, which is mainly due to an increase in false negatives. These false negatives were primarily due to highly expressed genes that show no differences in the detection rate. In summary, yes, top differential genes are detected with the binarized representation, given enough cells and samples.

**Rebuttal Figure 1** (Copied from previous work(9)): Plot of the logOR and logFC of the Cancer Atlas dataset. The x-axis represents the logFCs of each tested gene, and the y-axis represent the logORs for the same genes. The blue line shows the linear association between the logFC and logOR. The Spearman’s rank correlation coefficient is also shown in the plot.

6. In Figure 3c and 3d, the authors presented the classification result using SingleR. Can the authors comment on why there is a big drop in F1-score and accuracy for the shuffled datasets?

**Response:** This drop was due to a specific random split of the reference/target. We have now added new experiments, in which we repeat every experiment 10 times, each with a different random split of reference/target. Looking at the median F1-score of these 10 runs, we do not observe this drop in performance anymore. See answer to comment 7 of Reviewer 1 for a more elaborate discussion on the extended simulation experiment.

7. How does binarization affect the runtime and memory usage of the downstream analysis tasks presented in this work?

**Response:** We argue that specialized tools that operate on binarized representation need to be developed for this. As the storage requirements of binary data are significantly reduced compared to counts, we reason that runtime and memory usage of various analysis tasks can consequently be reduced considerably. We covered this in the discussion where we refer to BitClust(11).

**REVIEWER 4**

Reviewer #4: Bouland et al present a thought-provoking argument that scRNA-seq data analysis practitioners should more strongly and more regularly consider binarising scRNA-seq count data and then conduct downstream analysis on binary data (0, 1) rather than on the transcript/UMI count data usually used. The authors make some compelling arguments that binarisation should be used more often and the concise data analyses are effective in supporting their arguments. The crux is, they argue, that binarisation could/would/should make common analysis tasks more computationally efficient while sacrificing little in terms of accuracy or utility of the results obtained.

However, the paper in its current form completely lacks any description of methods used, so it is impossible to evaluate the rigour and accuracy of the data analysis results presented. Furthermore, there are several aspects of the argument/discussion that in my view need to be deepened and strengthened. If done, then I think this paper could make a useful Short Report, or similar, that would serve as a useful spur for discussion in the scRNA-seq data analysis community.

An alternative would be to shorten the manuscript and present it purely as an opinion/editorial article, in which case it could productively spur discussion in the field without requiring the depth and rigour of a Short Report or "full" paper.

As the comments below will show, I have a lot of issues with the paper in its current form. Overall, those issues are more related to the framing, discussion and presentation of the argument rather than the results, which I found interesting and informative. This paper prompted me to think further about this conundrum in scRNA-seq data analysis, but there is much that could be improved to make this paper a very valuable contribution to thinking in the field.

**Response**: We thank Dr. McCarthy for his comments. We prefer to keep the current format of the manuscript and as such, we have now: (1) added Supplementary Methods to describe the experiments in more detail, (2) added additional experiments to further support our claims, and (3) rewrote the discussion to strike a more balanced tone. We leave it to the discretion of the editor to decide whether the current format is the right format for our contribution.

Major Comments:

4.1⁃ No Methods section!

**Response:** This was indeed due to the chosen format. We have now added a detailed description of the methods as supplementary material.

4.2⁃ What about marker genes? In my experience, the actual expression level of marker genes used to define cell types is highly informative. Binarising data from marker genes is likely to remove the signal and diminish their utility. Ideally, the authors could provide some analyses addressing the issue of removing signal through binarising data - most obviously relevant in the case of marker genes, but other scenarios must also exist.

**Response:** In our previously published work (5), we show that using the differences in proportions of zeros and ones across cell types suffices to identify cell type specific genes (marker genes). We would also like to refer to our response to Comment 5 of Reviewer 3.

To further support previous results, we have now added a new analysis in which we use known brain cell type markers(12) to annotate cells from the *AD Data*(13) dataset based on their binarized representation. Cells were annotated based on the markers they express. Comparing the new annotations (based on the binary expression of the markers) to the original annotations (count-based), we obtained a median F1-score of 0.93 *(****SFig. 7****)*, indicating that the binary profile of marker genes is sufficient for cell annotation.

**Changes to the *results and discussion*:** “*Third, we evaluated the effect of binarization on cell annotation using (i) marker genes and (ii) classification methods. Using a set of known brain cell type markers(24), we annotated the binarized AD dataset(20) based on solely the detection of respective cell type markers (See Supplementary Methods). The annotations were compared to cell type labels that were originally assigned based on the markers' expression level (i.e., counts). We observed a high level of concordance between annotations as quantified by a median F1-score of 0.93. (SFig. 7). Additionally, we found that the visualization of the binarized expression of cell type markers to be highly similar to the visualization of their normalized expression in UMAP plots (Fig. 2e-h, SFig. 8).”*

**Changes in Supplementary Methods: “***To evaluate the use of marker genes with binarized scRNA-seq data, we annotated the cells from the ADData(43) dataset using markers from the BRETIGEA R-package(51). From the list of marker genes, we construct a one-hot-encoded marker matrix (*$M$*), where the columns represent cell types and the rows genes. When, gene* $g$ *is a marker for cell type* $c$*, then,* $m_{gc}=1$*, otherwise* $m_{gc}=0$*. Then, we subset the dataset, such that only known marker genes remain. Next, we calculate the Pearson’s correlation between the cell type vector (*$m_{c}$*) and the binarized expression of the cell (*$y_{j}$*). For every cell* $j$ *we get a measure of association (*$\varphi$*) with every cell type* $c$*. Higher values indicate higher association. As such, we annotated every cell as the cell type for which* $\varphi$ *is the highest. The approach was evaluated by comparing the annotations with the annotation from the original study. F1-scores for every cell type were calculated and the median F1-score was reported.”*

4.3⁃ The existence of ambient RNA ("background" RNA present from lysed cells and captured in droplets along with in-tact cells) poses substantial theoretical problems with binarisation. The authors claim that "binarization alleviate noise, as it is only subject to detection noise opposed to quantification and detection noise". However, under binarization ambient RNA will yield "detection" of a gene in a cell at the same level as true, endogenous (and possibly high) expression of a gene. In such cases, binarization will add detection noise compared to count-based quantification. This point warrants serious discussion in this paper (ideally supported by relevant analyses).

**Response:** It is true that ambient RNA will result in the detection of the respective gene. Consequently, we do agree that ambient RNA poses a challenge when studying individual cells. However, the downstream tasks that we considered are all at the population level and not at an individual cell level. As the distribution over the cells of ambient RNA is stochastic, it is likely that ambient RNA will be equally detected in different cell populations, and thus not affect the analyses tasks.

We did, however, remove the claim that binarization alleviates noise. We are now more specific, as we specify that binarization ignores count errors, but still suffers from detection errors caused by e.g. ambient RNA. In line with this, we also added a discussion surrounding ambient RNA.

**Changes to the *results and discussion*:** *“It has also been suggested that binarization alleviates noise(14) as it is insensitive to count errors. However, binarization remains sensitive to detection errors caused by, e.g., the presence of ambient RNA. Although, the presence of ambient RNA might not impact binary-based analyses on the cell population level, as ambient RNA is equally detected across cell populations due to its stochastic nature. Nonetheless, detection of ambient RNA(34) poses a challenge for binary representations when studying individual cells, and thus might require specialized methods to be developed.”*

4.4⁃ The authors claim: "standard count distribution models seem to fail in explaining the excess of zeros" (p1, l33) - this claim needs more discussion and evidentiary support. They cite a few papers supporting this view, but there are (at least as) many papers arguing the opposite - that sparse scRNA-seq count data is not zero-inflated (typically stated w.r.t. the negative binomial distribution).

**Response:** We believe we are on the same page here with Dr. McCarthy. We used this statement to highlight that there is a lot of discussion surrounding zeros in scRNA-seq data. Conclusions from our analyses do support the latter view, that sparse scRNA-seq data is *not* zero-inflated, if we consider the “right” cell population. To make this clearer, we moved the part in which we discuss zero-inflation and the origin of zeros from the discussion to the introduction. As such, the open remark that we make with “standard count distribution models seem to fail in explaining the excess of zeros” is immediately followed-up with more nuance and a solution.

Please see also our answer on Comment 2 of Reviewer #2 which addresses this point.

4.5⁃ Authors should avoid the term "dropouts" (p1 l39) - this is a muddied and unhelpful term. If they're talking about "observed zeros", then they should just use that term; if they're referring to other types (biological/technical) of zeros, then they should be specific. Jiang et al (2022) provide a very useful taxonomy of different types of observed zeros in scRNA-seq data - their definitions should better inform the discussion in this paper.

**Response:** Again, we fully agree with this statement. We regret that the original text did not faithfully reflect our thoughts, which resulted also in a similar comment by Reviewer 2. We refer to our answer to Comment 2 of Reviewer 2.

4.6⁃ Overall, I find the introduction (first two paras on p1) to be superficial and lacking. The start of the Discussion is much better, with mention of the Jiang et al and Sarkar and Stephens papers. A deeper, more mathematical introduction to the problem (binarization vs counts) that engages properly with these papers would drastically improve the introduction/framing of the paper. Unfortunately, the authors' claim that "this model [the Sarkar and Stephens model] is not suited for the observed zero inflation" is not strictly true. The hierarchical model proposed uses a Poisson model for the sampling variation of counts - with appropriate parameter definition in the hierarchical data generative model, the Sarkar-Stephens model can indeed account for "zero inflation" (if this exists - again, the claim of zero inflation only makes sense w.r.t. a specific model). I'm not convinced that the authors have fully absorbed the messages of the Sarkar and Stephens paper - as such, deeper and more rigorous discussion and treatment of that model is warranted here.

**Response:** We appreciate this comment and as such, we have now moved and extended the discussion on zeros to the introduction. In this part, we wanted to convey the message that any zero in scRNA-seq data contains valuable information about the expression, as explained by Sarkar and Stephens. We commented that the Poisson model on its own is not suited to explain the excess of zeros as the excess of zeros is due to biology as been pointed out by Sarkar and Stephens. Again, we refer to our answer to Comment 2 of Reviewer 2.

4.7⁃ The details of how scRATE are very important for assessing the accuracy of the enrichment results presented in Table 1 - without complete Methods we can't evaluate these results appropriately.

**Response:** In the newly added Supplementary Methods, we elaborate on these experiments.

**Changes in Supplementary Methods:** “*To test whether zero-inflation in scRNA-seq data can be explained by biological heterogeneity, we reasoned that a marker gene is a prime example of biological heterogeneity: it being highly expressed in a specific cell population while virtually absent in other cells. As such, we hypothesized that if zero-inflation is primarily explained by biological heterogeneity, marker genes should be zero-inflated. Using the same reasoning, a stably expressed gene should not be zero-inflated. We tested both hypotheses on two brain datasets(10x(44)and a Smart-Seq v2(59)). We selected two cell types and performed differential expression analyses between the cell types, using BDA(60). Next, we selected the top 100 most differentially expressed genes (sorted on PFDR), as well as the top 100 most stably expressed genes (sorted on smallest fold changes). Using scRATE(61), we fitted four different count distribution models (a Poisson, Negative-binomial and their zero-inflated counter parts) to all 200 genes individually. Using a leave-one-out cross validation test, we selected the best count distribution model for each gene, based on the best predictive accuracy. For every gene, we then know whether it is a marker gene or stably expressed, and whether it is zero-inflated or not. With a fisher exact test, we finally evaluate the association between zero-inflated/not zero inflated with marker/stable.*”

4.8⁃ Were scPred and SingleR pre-trained on binarised counts for this application, or pre-trained on raw counts? (Again, a Methods section should remove the need for confusion for the reader on such points.)

**Response:** For the binary-based analysis, scPred and SingleR were trained and evaluated on binary data. And for the count-based analysis, they were trained and evaluated on count data. The details of the classification experiments are now added to the Supplementary Methods.

4.9 ⁃ The authors claim that binarisation has the benefit of not needing to fuss about normalization/transformation. While that may be possibly true for the cell type identification and differential expression analyses shown in this paper, it is not true for any dimension reduction analyses (among other analyses one might do). For comparison, there is currently much debate on how to best apply flavours of TF-IDF (term frequency - inverse document frequency) normalization/transformation in the analysis of binary scATAC-seq data. I'm not at all persuaded that binarization of scRNA-seq data avoids complexities or concerns about normalization/transformation for many downstream analyses.

**Response:** We have now added additional experiments to the revised manuscript, showing that dimensionality reduction methods and data integration can be applied to binarized representations with comparable results to those obtained using counts. We do agree that this is not generalizable and there might be specific tasks for which counts are more suitable. For a more elaborate discussion on this topic, we refer to Comment 7 of Reviewer 2.

Minor Comments:

- the abbreviations "scRNA-seq" and "scRNA-seq" are both used in the abstract - choose one and be consistent

**Response:** Thanks, we now use consistent terms.

- p4 l23: "Sakar" should be "Sarkar"

**Response:** Corrected.

- More than once, in-text citations of papers contain misspellings of authors' names ("Sakar", "Jian") - please make sure these are correct.

**Response:** We apologize for these mistakes. It is corrected now.

Best wishes,

Davis J. McCarthy

**References**

1. Li,R. and Quon,G. (2019) ScBFA: Modeling detection patterns to mitigate technical noise in large-scale single-cell genomics data. *Genome Biol.*, **20**, 1–20.

2. Aran,D., Looney,A.P., Liu,L., Wu,E., Fong,V., Hsu,A., Chak,S., Naikawadi,R.P., Wolters,P.J., Abate,A.R., *et al.* (2019) Reference-based analysis of lung single-cell sequencing reveals a transitional profibrotic macrophage. *Nat. Immunol. 2019 202*, **20**, 163–172.

3. Alquicira-Hernandez,J., Sathe,A., Ji,H.P., Nguyen,Q. and Powell,J.E. (2019) ScPred: Accurate supervised method for cell type classification from single-cell RNA-seq data. *Genome Biol.*, **20**, 1–17.

4. Svensson,V. (2020) Droplet scRNA-seq is not zero-inflated. *Nat. Biotechnol.*, **38**, 147–150.

5. Bouland,G.A., Mahfouz,A. and Reinders,M.J.T. (2021) Differential analysis of binarized single-cell RNA sequencing data captures biological variation. *NAR Genomics Bioinforma.*, **3**.

6. Qiu,P. (2020) Embracing the dropouts in single-cell RNA-seq analysis. *Nat. Commun.*, **11**, 1–9.

7. Schmid,K.T., Höllbacher,B., Cruceanu,C., Böttcher,A., Lickert,H., Binder,E.B., Theis,F.J. and Heinig,M. (2021) scPower accelerates and optimizes the design of multi-sample single cell transcriptomic studies. *Nat. Commun. 2021 121*, **12**, 1–18.

8. Moignard,V., Woodhouse,S., Haghverdi,L., Lilly,A.J., Tanaka,Y., Wilkinson,A.C., Buettner,F., MacAulay,I.C., Jawaid,W., Diamanti,E., *et al.* (2015) Decoding the regulatory network of early blood development from single-cell gene expression measurements. *Nat. Biotechnol. 2015 333*, **33**, 269–276.

9. Bouland,G.A., Mahfouz,A. and Reinders,M.J.T. (2021) Differential dropout analysis captures biological variation in single-cell RNA sequencing data. *bioRxiv*, 10.1101/2021.02.01.429187.

10. Yazar,S., Alquicira-Hernandez,J., Wing,K., Senabouth,A., Gordon,M.G., Andersen,S., Lu,Q., Rowson,A., Taylor,T.R.P., Clarke,L., *et al.* (2022) Single-cell eQTL mapping identifies cell type-specific genetic control of autoimmune disease. *Science*, **376**.

11. González-Alemán,R., Hernández-Castillo,D., Rodríguez-Serradet,A., Caballero,J., Hernández-Rodríguez,E.W. and Montero-Cabrera,L. (2020) BitClust: Fast Geometrical Clustering of Long Molecular Dynamics Simulations. *J. Chem. Inf. Model.*, **60**, 444–448.

12. McKenzie,A.T., Wang,M., Hauberg,M.E., Fullard,J.F., Kozlenkov,A., Keenan,A., Hurd,Y.L., Dracheva,S., Casaccia,P., Roussos,P., *et al.* (2018) Brain Cell Type Specific Gene Expression and Co-expression Network Architectures. *Sci. Reports 2018 81*, **8**, 1–19.

13. Grubman,A., Chew,G., Ouyang,J.F., Sun,G., Choo,X.Y., McLean,C., Simmons,R.K., Buckberry,S., Vargas-Landin,D.B., Poppe,D., *et al.* (2019) A single-cell atlas of entorhinal cortex from individuals with Alzheimer’s disease reveals cell type-specific gene expression regulation. *Nat. Neurosci.*, **22**, 2087–2097.

**Second round of review**

**Reviewer 2**

Overall, the authors have drastically improved the manuscript. Here are a number of points that still might require some clarification.

1. In the "Background" paragraph, I think it is already worth mentioning as motivation, why representing such datasets as binary is useful as the "preferred data representation": space savings! Maybe it's obvious, but it is not mentioned until much later.

2. "shallow sequencing of many cells is more cost effective than deep sequencing of a few cells". I don't think this is always the case, because it depends on the biological question. Similarly "measuring more cells increases the overall power"; yes, on average, it does. But it may not increase the power to detect certain physiological processes (e.g., low to medium expressed genes) barely at all in some rarer populations, so I'm generally uncomfortable about these statements that 'more is better'. I would hope for a more balanced representation of the situation.

3. "overall power to detect genes" --> I think you mean here "overall power to detect DE genes"

4. Figure 1C .. it may be worth pointing out that the Pearson coefficient you are calculating is between something continuous and binary, which means it is a point-biserial correlation. Perhaps a Supplementary Figure that would be of interest in connection with Figure 1C/D is the distribution of p for each dataset. It seems to me what is more interesting than Figure 1D is how *low* the correlation becomes when the q values are high, not the correlation of q with p.

5. For the demonstration that dimension reduction works well on binarized data, one plot that could be particularly useful are those like Fig 5. in the Becht paper (DOI: 10.1038/nbt.4314); because there you could show directly that the distances are preserved between count-based and binarized. I find the analyses that claim "visually very similar" a little hard to digest.

6. While I appreciate the expanded analysis of simulated scRNA-seq for differential state detection, the results are still hard to access. I guess the plan is a Short Report, which makes this all difficult, because each of these analyses is interesting and could be expanded on. From Supp Fig 11, I'm not sure that F1 score is what typically people would look at here (usually, you would plot power versus achieve false discovery rate), because with F1s you don't get a sense of whether the tests used are calibrated. In any case, Supp Fig 10 shows that in most cases, having the full counts buys you better performance. The conclusion in the paper was that "F1-scores .. were very similar", which is also true, but hides the reality that very "similar" here means that counts are still generally useful to have. And here, it would benefit from a few extra descriptions. In the top left panel, it has 5 vs 5 and 1000 cells; does that mean 100 cells per sample? If so, that seems already like a decently-powered situation. What if you have fewer than 5 experimental units (replicates) and rarer cell types (fewer than 100 cells per sample)? There is a big space to cover and the take-home conclusion of "F1 scores were similar" doesn't seem to fully represent it.

7. "ambient RNA is equally detected across cell populations due to its stochastic nature": is this really true? I thought the whole problem with ambient RNA was that some populations are more affected than others.

**Authors Response**

**Point-by-point responses to the reviewers’ comments:**

**Reviewer #2**: Overall, the authors have drastically improved the manuscript. Here are a number of points that still might require some clarification.

1. In the "Background" paragraph, I think it is already worth mentioning as motivation, why representing such datasets as binary is useful as the "preferred data representation": space savings! Maybe it's obvious, but it is not mentioned until much later.

*Thanks, it is indeed worth mentioning this already in the background. Changed in introduction: Provided that a binarized data representation has the potential to reduce required computational resources considerably, and as scRNA-seq datasets are becoming increasingly bigger and sparser, we wondered if binary should be the preferred data representation for other tasks.*

2. "shallow sequencing of many cells is more cost effective than deep sequencing of a few cells". I don't think this is always the case, because it depends on the biological question.

*Although we based this statement on conclusions drawn by Zhang et al (16) (given a fixed budget, they found that, shallow sequencing of many cells is more cost effective than deep sequencing of a few cells when estimating the true underlying distribution of a given gene), we do agree it will depend on the biological question at hand.*

*Changes to manuscript: Note that this trend of measuring more cells per dataset outweighs improved chemistry over time, and thus still results in sparser datasets. It is likely that this trend will continue over the next years as, for many biological questions, shallow sequencing of many cells is more cost effective than deep sequencing of a few cells (16).*

Similarly "measuring more cells increases the overall power"; yes, on average, it does. But it may not increase the power to detect certain physiological processes (e.g., low to medium expressed genes) barely at all in some rarer populations, so I'm generally uncomfortable about these statements that 'more is better'. I would hope for a more balanced representation of the situation.

3. "overall power to detect genes" --> I think you mean here "overall power to detect DE genes"

*In hindsight, the terminology we used “power to detect genes” is confusing. With this statement, we meant that the estimator of the probability that a gene is expressed is more accurate when more cells are being measured. But indeed, it is also true that measuring more cells increases the overall power to detect DE genes (also concluded by Schmid et al. (17)).*

*Changed to manuscript: Moreover, by measuring more cells we can better estimate the probability whether a gene is expressed, and the overall power to detect differentially expressed genes in a given dataset increases (17).*

4. Figure 1C .. it may be worth pointing out that the Pearson coefficient you are calculating is between something continuous and binary, which means it is a point- biserial correlation.

*Changes to manuscript: Using ~1.5 million cells from 56 datasets, we observed on average a strong point-biserial correlation (Pearson correlation coefficient p = 0.93) between the normalized expression counts of a cell and its respective binarized variant.*

Perhaps a Supplementary Figure that would be of interest in connection with Figure 1C/D is the distribution of p for each dataset. It seems to me what is more interesting than Figure 1D is how *low* the correlation becomes when the q values are high, not the correlation of q with p.

*We have added a supplemental figure (Supplement Figure 1, shown below) in which the distribution of p is visualized for every dataset. Changes to manuscript: Using ~1.5 million cells from 56 datasets, we observed on average a strong point-biserial correlation (Pearson correlation coefficient p = 0.93) between the normalized expression counts of a cell and its respective binarized variant, although differences between datasets exist (SFig 1).*

*Additional file 1: Fig S1: The distributions of correlation coefficients between the binarized and count-based expressions of every cell (p, x-axis) within each dataset (y- axis). The datasets are grouped by technology.*

5. For the demonstration that dimension reduction works well on binarized data, one plot that could be particularly useful are those like Fig 5. in the Becht paper (DOI: 10.1038/nbt.4314); because there you could show directly that the distances are preserved between count-based and binarized. I find the analyses that claim "visually very similar" a little hard to digest.

*We have added a similar figure to the supplements (Supplement Figure 5, shown below).*

*Changes to manuscript: This was confirmed quantitatively, as the pairwise distances between cells based on the binary-based UMAPs were highly correlated with the pairwise distances from the count-based UMAP (r ≥ 0.73, SFig. 5).*

*Additional file 1 Fig. S5: Association of pairwise eucledian distances between cells from count based UMAP with A) binary-PCA based UMAP, B) JaccardEV based UMAP and C) scBFA based UMAP. First, 5,000 cells were randomly sampled, between which the pairwise eucledian distance was calculated based on the different UMAPs. Based on these pair-wise distances (n = 12,497,500) the pearson correlation was calculated. For plotting 10,000 points were randomly sampled from total number of calculated pair-wise distances.*

6. While I appreciate the expanded analysis of simulated scRNA-seq for differential state detection, the results are still hard to access. I guess the plan is a Short Report, which makes this all difficult, because each of these analyses is interesting and could be expanded on. From Supp Fig 11, I'm not sure that F1 score is what typically people would look at here (usually, you would plot power versus achieve false discovery rate), because with F1s you don't get a sense of whether the tests used are calibrated. In any case, Supp Fig 11 shows that in most cases, having the full counts buys you better performance. The conclusion in the paper was that "F1-scores .. were very similar", which is also true, but hides the reality that very "similar" here means that counts are still generally useful to have. And here, it would benefit from a few extra descriptions. In the top left panel, it has 5 vs 5 and 1000 cells; does that mean 100 cells per sample? If so, that seems already like a decently-powered situation. What if you have fewer than 5 experimental units (replicates) and rarer cell types (fewer than 100 cells per sample)? There is a big space to cover and the take-home conclusion of "F1 scores were similar" doesn't seem to fully represent it.

*In addition to the F1-score we report the FPR (Additional file 1: Fig S14) and FNR (Additional file 1: Fig S15). Using these three metrics in the current analysis, we do believe we have showed the limitations of binarization in this type of analysis, and we regret that the nuance of our conclusion was not conveyed properly. Over the 96 different settings, the F1 scores are similar. However, with lower sample sizes and fewer cells, counts are performing better than binarized counts. On the other hand, with more samples and more cells binarized counts perform better. Our simulation includes a comparison of 5 vs 5 samples, each with 100 cells, this is already the threshold at which counts perform clearly better and are still useful to have. Going lower in number of cells shows the same. Consequently, we do not believe that going below 5 samples with even less cells will add anything, as it will show the same again.*

*Changes in manuscript: All together, these result show that most of the information is indeed captured in the binary representation, only when genes have a high detection rate (>0.9) or when the number of cells per sample becomes low, then, changes in expression are not reflected in the binary representation and, consequently, information from counts is needed.*

*Changes in caption of Additional file: Figure S13: F1-score of 960 simulated datasets indicating the accuracy of detecting differentially expressed genes in simulated pseudobulk data when either count or binarized data are used. The x-axis represents the fraction of simulated differentially expressed genes. The y-axis represents the F1- score. The top-left panel represents a comparison of 5 vs. 5 samples in a simulated dataset of 1.000 cells, meaning that each sample was comprised of 100 cells. E.g in the bottom left panel each sample was comprised of 10 cells.*

7. "ambient RNA is equally detected across cell populations due to its stochastic nature": is this really true? I thought the whole problem with ambient RNA was that some populations are more affected than others.

*We looked a bit better into this, and the reviewer is right (eg. Yang et al [34]). We thus excluded this statement and refrain to mention problems with ambient RNA. Changes in manuscript: However, binarization remains sensitive to detection errors caused by, e.g., the presence of ambient RNA. Consequently, detection of ambient RNA(34) poses a challenge for binary representations when studying individual cells, and thus might require specialized methods to be developed.*
